# Impacts of Nitrogen Deficiency on Wheat (*Triticum aestivum* L.) Grain During the Medium Filling Stage: Transcriptomic and Metabolomic Comparisons

**DOI:** 10.3389/fpls.2021.674433

**Published:** 2021-08-04

**Authors:** Yanjie Wang, Demei Wang, Zhiqiang Tao, Yushuang Yang, Zhenxian Gao, Guangcai Zhao, Xuhong Chang

**Affiliations:** ^1^Center for Crop Management and Farming System, Institute of Crop Sciences, Chinese Academy of Agricultural Sciences/Key Laboratory of Crop Physiology and Ecology, Ministry of Agriculture, Beijing, China; ^2^Wheat Research Center, Shijiazhuang Academy of Agricultural and Forestry Sciences, Shijiazhuang, China

**Keywords:** nitrogen deficiency, wheat (*Triticum aestivum*), RNA-Seq, metabolome, photosynthesis, amino acids, sugars, cell wall

## Abstract

Nitrogen (N) supplementation is essential to the yield and quality of bread wheat (*Triticum aestivum* L.). The impact of N-deficiency on wheat at the seedling stage has been previously reported, but the impact of distinct N regimes applied at the seedling stage with continuous application on filling and maturing wheat grains is lesser known, despite the filling stage being critical for final grain yield and flour quality. Here, we compared phenotype characteristics such as grain yield, grain protein and sugar quality, plant growth, leaf photosynthesis of wheat under N-deficient and N-sufficient conditions imposed prior to sowing (120 kg/hm^2^) and in the jointing stage (120 kg/hm^2^), and then evaluated the effects of this continued stress through RNA-seq and GC-MS metabolomics profiling of grain at the mid-filling stage. The results showed that except for an increase in grain size and weight, and in the content of total sugar, starch, and fiber in bran fraction and white flour, the other metrics were all decreased under N-deficiency conditions. A total of 761 differentially expressed genes (DEGs) and 77 differentially accumulated metabolites (DAMs) were identified. Under N-deficiency, 51 down-regulated DEGs were involved in the process of impeding chlorophyll synthesis, chloroplast development, light harvesting, and electron transfer functions of photosystem, which resulted in the SPAD and Pn value decreased by 32 and 15.2% compared with N-sufficiency, inhibited photosynthesis. Twenty-four DEGs implicated the inhibition of amino acids synthesis and protein transport, in agreement with a 17–42% reduction in ornithine, cysteine, aspartate, and tyrosine from metabolome, and an 18.6% reduction in grain protein content. However, 14 DEGs were implicated in promoting sugar accumulation in the cell wall and another six DEGs also enhanced cell wall synthesis, which significantly increased fiber content in the endosperm and likely contributed to increasing the thousands-grain weight (TGW). Moreover, RNA-seq profiling suggested that wheat grain can improve the capacity of DNA repair, iron uptake, disease and abiotic stress resistance, and oxidative stress scavenging through increasing the content levels of anthocyanin, flavonoid, GABA, galactose, and glucose under N-deficiency condition. This study identified candidate genes and metabolites related to low N adaption and tolerance that may provide new insights into a comprehensive understanding of the genotype-specific differences in performance under N-deficiency conditions.

## Introduction

Wheat (*Triticum aestivum* L.) is one of the most important cereal crops and the second most widely grown crop in the world. Wheat grain consumption accounts for >40% of the global human diet (Shewry, 2009), and it is mainly used for food production including for bread, cakes, biscuits, pasta, and noodles (Zörb et al., 2018). Mature wheat grain is rich in carbohydrates (mainly represented by starch accounting for 60–70% of dry weight) and has a higher protein content (accounting for 10–18% of endosperm dry weight) compared with other major cereals, such as rice (*Oryza sativa*), maize (*Zea mays*), rye (*Secale cereale*), and millet (*Pennisetum glaucum*) (Ashraf, 2014).

Nitrogen (N) is a necessary nutrient required for crop growth and development. Wheat grain yield and quality depend upon substantial inputs of N. Moreover, N plays the most important role in determining protein content, dough quality, and processing characteristic (Barraclough et al., 2010; Xue et al., 2016). However, wheat is the major crop with the lowest nitrogen use efficiency (NUE), and only 30–35% of applied N fertilizer could be absorbed (Raun and Johnson, 1999; Gaju et al., 2011). The excessive application of N fertilizer frequently occurs during production, and a two-fold increase in food production required more than a seven-fold increase in N fertilizer application over the past 40 years, producing immense waste and pollution (Hirel et al., 2007). Therefore, understanding the mechanisms of how N influences the yield and quality of wheat grain is of great significance for N fertilizer control.

Wheat has evolved many adaptive strategies to deal with N deficiency under field conditions. Examples of this included morphological changes in shoot and leaf development (Curci et al., 2017; Wang et al., 2019) and root architecture (Xue et al., 2014; Lv et al., 2020). Physiological adaptations included altering photosynthesis and enzyme activity related to N assimilation, such as glutamine synthetase, glutamate synthase, nitrate reductase, Rubisco, H^+^-ATPase (Li et al., 2013; Lv et al., 2020), and decreased malondialdehyde content (Guo et al., 2014). Biochemical responses such as altered phytohormone signaling in roots relative to indole-3-acetic acid (IAA), cytokinin (CTK), gibberellin (GA3), and jasmonic acid (JA) concentrations stimulated by low-nitrogen stress (Lv et al., 2020). It has also been observed that wheat increased gene expression levels of nitrate transporters, glutamine synthetase, and those controlling NUE, such as *NRT1* and *NRT2* (Guo et al., 2014), *TaVRN-A1* (Lei et al., 2018), *TabZIP1*, and *TaPIMP1TF* (Mahmoud et al., 2020), which could enhance the uptake of N and stimulate root development. In particular, *TaVRN-A1* conferred a winter wheat grain yield increase of 18.1% in the field under N-starvation conditions (Lei et al., 2018).

Diverse “omics” approaches such as RNA sequencing (RNA-seq) of the transcriptome and metabolomics profiling have been increasingly applied to detect the genetic mechanisms responsible for plant biotic and abiotic stresses. RNA sequencing has been applied to numerous wheat tissues under different stress conditions such as wheat seedling and roots under low pH (Hu et al., 2018), salinity stress (Amirbakhtiar et al., 2019), as well as upon invasion by arbuscular mycorrhizal fungi (Li et al., 2018a), wheat grain response to vernalization (Feng et al., 2016), drought stress (Ma et al., 2017), and heat stress during the filling stage (Rangan et al., 2020). Together, these studies provided a vital molecular-level understanding of the stress mechanism for wheat grain development, yield, and quality formation. Metabolomics profiling has been applied on crops to detect the metabolic changes responsive to abiotic stresses, including barley (*Hordeum vulgare*) under phosphorus-deficiency conditions (Huang et al., 2008), rice (*O. sativa*) under high temperature conditions (Yamakawa and Hakata, 2010), maize (*Z. mays*) subjected to drought stress (Witt et al., 2012), soybean in response to low-N tolerance (Li et al., 2018b), and durum wheat (*Triticum turgidum* Desf.) under water stress conditions (Vergara-Diaz et al., 2020). The integration of transcriptomic and metabolomic approaches is being increasingly applied to different crops to reveal the molecular mechanism of resistance to environmental stresses based on existing genetic, physiological, and morphological data (Bowne et al., 2011). These synergistic efforts have been applied to the detection of tolerance mechanisms for wild soybean seedling roots under low N stress conditions (Liu et al., 2020a), the identification of candidate genes that might be active in oat adaptation to P deficiency (Wang et al., 2018), the discovery of vital genes and metabolic changes in wheat seedlings in response to cold stress (Zhao et al., 2019), uncovering malting quality regulatory networks of barley under drought stress conditions (Hong et al., 2020), and carbon and N metabolism of rice in response to high N (Xin et al., 2019).

Despite the importance of N in wheat cultivation, limited research has been conducted concerning the effect of different N-application rates at the seedling stage and its impact on grain maturation and production. The last two decades have delivered tremendous progress in understanding the yield, quality, genetic regulations of NUE, as well as the morphological, physiological, and biochemical effects of N deficiency. However, these studies mainly focused on roots and leaves in bread wheat seedlings (Wang et al., 2019; Xin et al., 2020), or only investigated the transcriptome response to N-starvation (Sultana et al., 2020) or metabolome response to high-N condition (Zhen et al., 2016) of bread wheat in filling grain. Furthermore, the variation in gene expressions and metabolite levels at the medium stage of grain filling changed to the greatest degree and closely influenced the yield and quality of grain (Zhen et al., 2016; Henry et al., 2018). Therefore, we performed gene expression and metabolite profiling to evaluate the effect of N-sufficient and N-deficient conditions imposed during the seedling and jointing stages and their effect on grain filling and maturation. The aims were (i) to identify the differentially expressed genes (DEGs) related to photosynthesis of wheat grain in response to continuous N deficiency stress, (ii) to discover the DEGs and differentially accumulated metabolites (DAMs) linked to stress tolerance that were triggered by continuous N-deficiency stress, and (iii) to analyze the molecular basis of changes in thousand-grains weight (TGW) and protein content affected by N, especially amino acid and sugar metabolism. The findings from this study will provide a foundation for the guidance of N fertilizer application to optimize wheat grain yield and quality.

## Materials and Methods

### Wheat Materials and Field Experiment Design

Shiluan 02-1 was the selected wheat variety for the studies. Shiluan 02-1 is a typical high-gluten bread wheat cultivar and has a sensitive response to N fertilizer (unpublished), which was released in China in 2007 and is widely cultivated in north China. The experiments were performed in large growth containers (each 18 m^3^ cement pools, 3 m length ^*^ 3 m width ^*^ 2 m depth) located in Zhao county, Hebei province, during the continuous 2016–2018 wheat-growing season. The organic content of 0–40 cm-deep soil was 8.78 g/kg, total N was.8 g/kg, available N was 56.94 mg/kg, rapidly available phosphorus (P_2_O_5_) was 32.45 mg/kg, and rapidly available potassium (K_2_O) was 92 mg/kg with a pH of 7.8.

We selected urea (NH_2_)_2_CO as the N fertilizer and designed two experimental groups: a control group of 240 kg/hm^2^ urea (N-sufficient; Nck), and a treatment group of 0 kg/hm^2^ (N-deficient; N0). The N application time of the Nck group occurred prior to sowing (120 kg/hm^2^) and jointing stage (120 kg/hm^2^). P_2_O_5_ 172.5 kg/hm^2^ and K_2_O 112.5 kg/hm^2^ were uniformly applied before sowing. A total of six cement pools were used in the experiment, each group comprised three cement pools, which represented three biological replicates. The planting density was 225 ^*^ 10^4^ plants/hm^2^, the typical rate in the North China Plain (NCP) (2,025 total plants in each plot). The plants were sown by hand in 10 rows, and the row spacing was 20 cm. The plants were irrigated with 900 m^3^/hm^2^ for overwintering, 750 m^3^/hm^2^ water at the jointing and flowering stages, respectively. In the 2016–2018 growing season, the average annual precipitation was 278 mm, the average annual sunshine duration was 1,611 h, and the average annual temperature was 9.75°C at the experiment location. The grains at 25 days post anthesis (DPA) were collected and immediately frozen in liquid N_2_, and then stored at −80°C prior to RNA and metabolites extraction.

### Photosynthetic Evaluation, Grain Yield, and Quality Testing

At 25-DPA, the net photosynthetic (Pn) rate of flag leaves was measured with Li-6400XT Portable Photosynthesis System (LI-COR Biosciences, Lincoln, NE, United States) at 11:00 a.m. The chlorophyll concentration of the flag leaves was measured with SPAD (soil and plant analyzer development)-502 Plus Chlorophyll Meter (Konica Minolta, Japan), known as SPAD value at the same time. The length and width of the flag leaves were also measured at 25-DPA.

The plant height and spike length were evaluated after harvesting. The mature grains from each plot were harvested using a mini-Vogel machine. The grain yield per plot (kg/hm^2^), spike number per unit area (No. spikes/hm^2^), grain number per spike (No. grains/spike), TGW (g), grain length, and grain width of mature grain were measured. Grain protein fractions including albumin, globulin, gliadin, and glutenin were extracted with a continuous extraction method, which, together with grain total protein content (5.7% N, 14% moisture basis), were all determined by the Kjeldahl N determination method (Kjeldahl, 1883) using the Automatic Kieldahl Apparatus (K9860, Hanon, Jinan China). The content of total sugar, starch, and fiber content in the bran fraction (including the episperm and aleurone layer) and white flour (endosperm and embryo) of matured grain were measured. Total sugar in matured grain was measured as well, which was used to estimate the ratio of carbon to N. The anthrone-sulfuric acid colorimetric assay was used to determine the total sugar (carbohydrate) content (Laurentin and Edwards, 2003). The anthronecolorimetric method was used to estimate starch and fiber content (Clegg, 1956). All metrics were measured using the mean of three replicate experiments.

### RNA Extraction, Library, and Sequencing

Total RNA was extracted from wheat grain of 25 DPA using the mirVana miRNA Isolation Kit (Ambion-1561, Ambion, Austin, TX, United States) following the protocol of the manufacturer. The quality and concentration of extracted RNA were determined using the Agilent 2100 Bioanalyzer (Agilent, Santa Clara, CA, United States) and NanoDrop2000 spectrophotometer (Thermo Fisher Scientific, Waltham, MA, United States), respectively. The samples with RNA integrity number (RIN) ≥ 7 were subjected to the subsequent analysis. RNA libraries were constructed using TruSeq Stranded mRNA LT Sample Prep Kit (Illumina, San Diego, CA, United States) according to the instructions of the manufacturer. Then, six independent grain cDNA libraries were sequenced on the Illumina HiSeq X Ten sequencing platform (Shanghai OE Biotech. Co., Ltd. Shanghai, China) and 150 bp paired-end reads were generated.

### Quality Control and Differentially Expressed Genes Analysis

A total of 310.49 million (97.21%) clean reads were obtained using the NGS QC Toolkit (Patel and Jain, 2012). Meanwhile, a total of 44.46 Gb clean data were obtained with an average of 7.41 Gb per sample. The GC content ranged from 51.25 to 51.89%. Among all of the reads, more than 93.51% had Phred-like quality scores at the Q30 level (an error probability of 0.1%); 92.19–93.22% of the clean reads obtained from each sample were mapped to reference sequences using hisat2 (Kim et al., 2015), and 83.11–85.3% were uniquely matched (Table 1). Fragments Per kb Per Million Reads (FPKM) (Trapnell et al., 2010) values for each gene were calculated using Cufflinks (Roberts et al., 2011), and DEGs were identified using the DESeq (Anders and Huber, 2012) R package function of estimated Size Factors and the nbinom Test. *P*-value < 0.05 and fold change >2 or fold change <0.5 (|log_2_FC| > 1) were set as the threshold for significantly differential expression. Hierarchical cluster analysis of DEGs was performed to explore gene expression patterns. Gene Ontology (GO) (Young et al., 2010) and Kyoto Encyclopedia of Genes and Genomes (KEGG) (Kanehisa et al., 2008) enrichment analyses of DEGs were respectively performed using R based on the hypergeometric distribution. The calculated result will return a *P*-value of enrichment significance, and a smaller *P*-value indicates that DEGs are enriched in the GO or KEGG pathway.

**Table 1:** Transcriptome sequencing data quality and genome mapping results.

**Summary**	**N0-1**	**N0-2**	**N0-3**	**Nck-1**	**Nck-2**	**Nck-3**
Total raw reads (nt, million)	53.43	52.47	54.05	53.90	53.16	52.40
Total clean reads (nt, million)	51.90	51.04	52.45	52.44	51.61	51.04
Total clean bases (Gb)	7.47	7.31	7.50	7.50	7.38	7.30
Total mapped reads (nt, million)	48.36	47.39	48.87	48.35	48.11	47.45
Uniquely mapped (nt, million)	44.26	43.37	44.61	43.59	44.02	43.29
Q30 (%)	93.52	93.93	93.51	93.86	93.76	94.04
GC content (%)	51.78	51.89	51.58	51.25	51.83	51.60

*nt, nucleotides*.

### Quantitative Real-Time PCR Analysis

Quantification was performed using a two-step reaction process consisting of reverse transcription (RT) and PCR. Each RT reaction consisted of 0.5 μg RNA, 2 μl of 5 × *TransScript* All-in-one *SuperMix* for qPCR and 0.5 μl of gDNA Remover in a total volume of 10 μl. Reactions were performed in a *GeneAmp*® PCR System 9700 (Applied Biosystems, Waltham, MA, United States) for 15 min at 42°C, 5 s at 85°C. The 10 μl RT reaction mixture was then diluted 10-fold in nuclease-free water and kept at −20°C. Real-time PCR was performed using *LightCycler*® 480 II Real-time PCR Instrument (Roche, Basel, Switzerland) with 10 μl PCR reaction mixture that included 1 μl of cDNA, 5 μl of 2 × *PerfectStart*™ Green qPCR SuperMix, 0.2 μl of 10 μM forward primer, 0.2 μl of 10 μM reverse primer, and 3.6 μl of nuclease-free water. Reactions were incubated in a 384-well optical plate (Roche, Swiss) at 94°C for 30 s, followed by 45 cycles of 94°C for 5 s and 60°C for 30 s. Each sample was run in technical triplicate for analysis. At the end of the PCR cycles, a melting curve analysis was performed to validate the specific generation of the expected PCR product. The primer sequences are shown in Supplementary Table 1. The expression levels of mRNAs were normalized to endogenous reference gene β*-actin* (forward primer: 5′-TCCAATCTATGAGGGATACACGC-3′, reverse primer: 5′-TCTTCATTAGATTATCCGTGAGGTC-3′) and were calculated using the 2^−ΔΔCt^ method (Livak and Schmittgen, 2001). The RT-qPCR data were represented as log_2_ Fold change (value N_0_/N_ck_) derived from normalized expression level from three technologic replicates and further compared with RNA-seq results (RPKM N_0_/N_ck_) by means of Pearson correlation analysis with Data Processing System (DPS) (Tang and Zhang, 2013).

### Grain Metabolites Extraction, GC-MS Profiling

Six sampling replicates of grains from individual plants grown under N0 and Nck conditions were sampled, and each biological replicate comprised two sampling replicates. Sixty milligrams from each sample was accurately weighed and extracted in 360 μl of cold methanol and 40 μl of 2-chloro-l-phenylalanine (0.3 mg/ml), as well as 200 μl of CH_4_Cl_3_ and 400 μl of water. Quality control (QC) sample was prepared by mixing aliquots of all the samples into a pooled sample. The samples were placed at ambient temperature for 30 min before gas chromatography-mass spectrometry (GC-MS) analysis. The derivatized samples were analyzed on an Agilent 7890B GC system coupled to an Agilent 5977A MSD system (Agilent Technologies Inc., Sta. Clara, CA, United States). Metabolites were annotated through the Fiehn or NIST database. The values of the data matrix were converted to log_2_, 0 was replaced with 0.000001, and then multivariate statistical analyses were performed based on N0 and Nck metabolites with the aid of SIMCAP v14.0 (Umetrics, Umeå, Sweden), namely, principal component analysis (PCA), partial least squares discriminant analysis (PLS-DA), orthogonal partial least squares discriminant analysis (OPLS-DA), and response permutation test (RPT) analysis (Supplementary Figure 1). The cumulative interpretation rate R^2^*X* (0.461) and prediction rate Q^2^ (0.151) of the PCA model are relatively high, which can explain and predict the difference between the two groups. The transcriptomic and metabolomic experiments were conducted by Oebiotech, Shanghai, China (http://www.oebiotech.com/).

### Identification of Differentially Accumulated Metabolites

Differentially accumulated metabolites were determined according to the following criteria: VIP > 1 (variable importance in the projection values obtained from the OPLS-DA model, the larger VIP, the greater contribution to grouping), *P* < 0.05 (the result of Student's *t*-test, evaluates the difference between the two groups of samples), and FC > 1 represented up-regulated or FC <1 represented down-regulated (FC is fold change, represents the ratio of the average content of the different metabolite in the two groups, N0/Nck).

### Principal Component Analysis and Statistical Analysis

Principal component analysis was examined using the R software (version 3.2.2). Duncan's Multiple Range Test (DMRT) on the DPS statistical software was performed to examine significant differences of physiological traits and gene expression between N0 and Nck treatments (Tang and Zhang, 2013). *P* < 0.05 was considered to be significant, and *P* < 0.01 was considered to be extremely significant.

## Results

### Comparison of Photosynthesis, Grain Yield, and Grain Quality Under N-Deficient and N-Sufficient Conditions

**Figure 1:**
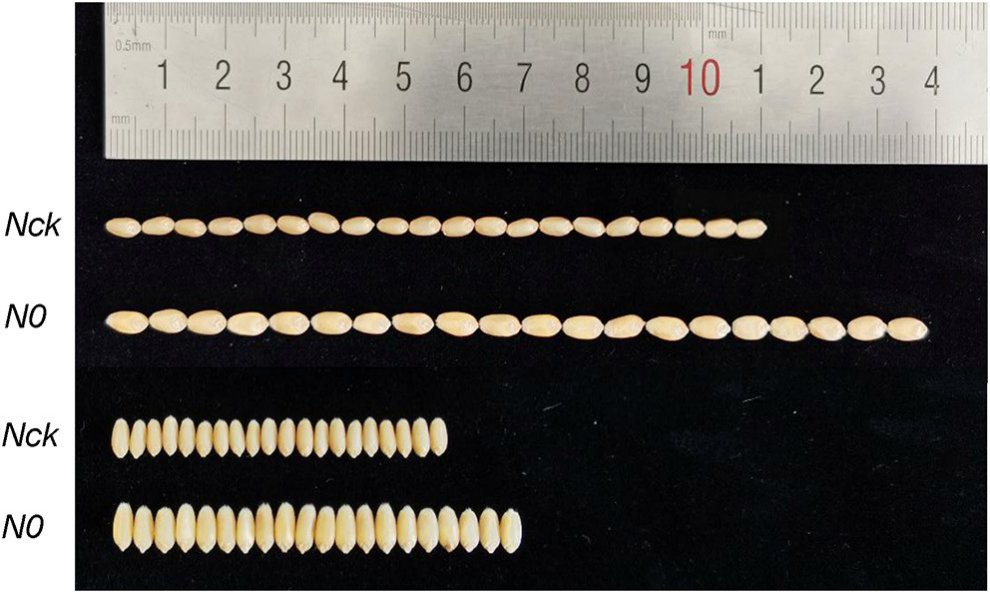
Comparison of mature grain length and width with N0 and Nck treatments.

The N-sufficient condition was imposed prior to sowing (120 kg/hm^2^) and in the jointing stage (120 kg/hm^2^), and there was no N fertilizer application under the N-deficient condition. At 25 days of anthesis, the photosynthesis and SPAD value, leaf length, and width of the flag leaf were extremely significantly decreased (*P* < 0.01) under the N0 conditions compared with those of the N sufficient conditions. After harvesting, grain yield (No. grains/spike, No. spikes 10^4^/hm^2^) and total protein content was also extremely significantly reduced (*P* < 0.01) under the N0 conditions. In general, grain yield, No. grains/spike, flag leaf size, chlorophyll content, grain total protein, and gliadin content under N0 conditions were extremely significantly affected (*P* < 0.01) by N stress, whereas No. spikes/hm^2^ and albumin content were significantly affected (*P* < 0.05). However, the TGW, grain length, and grain width of N0 exhibited 12.5, 3.3, and 4.98% increases compared with those of Nck grain (Table 2; Figure 1). Concerning yield indicators, wheat grain subjected to the N0 conditions showed a 19.3% reduction in grain yield, a 25.6% decrease in No. grains/spike, and a 3.7% decrease in No. spikes/hm^2^ on average compared with the Nck conditions. For plant growth, N0 plant morphology showed shorter plant height (8.6%), smaller flag leaf length (28.1%), and width (20%) than in the Nck-group. The Pn and SPAD values of flag leaf grown under the N0 conditions were also 15.2 and 32% lower than in Nck.

**Table 2:** Comparison of yield, plant growth, photosynthesis, and quality indicators of wheat grown under N0 and Nck conditions.

	**Grain yield**				**Grain character**		**Leaf photosynthesis**	
**Treatment**	**Grain yield** **(kg/hm** ^**2**^ **)**	**No. grains/** **spike**	**No. spikes** **10** ^**4**^ **/hm** ^**2**^	**TGW (g)**	**Grain length** **(mm)**	**Grain** **width (mm)**	**Pn** **(μmol·m** ^**–2**^ **·** **s** ^**–1**^ **)**	**SPAD**
N_0_	6,677.26 ± 67.05^**^	31 ± 1^**^	38.69 ± 1.21^**^	37.11 ± 0.23^**^	6.11 ± 0.03^**^	3.15 ± 0.05^**^	13.75 ± 0.54^**^	34.23 ± 1.99^**^
N_ck_	8,278.60 ± 120.75	41.67 ± 1.53	40.17 ± 1.32	32.97 ± 1.02	5.92 ± 0.05^**^	3.00 ± 0.03	16.21 ± 1.11	50.3 ± 0.5
	**Plant growth**				**Protein**			
**Treatment**	**Plant height** **(cm)**	**Flag leaf** **length (cm)**	**Flag leaf** **width (cm)**	**Total protein** **(%)**	**Albumin** **(%)**	**Globulin** **(%)**	**Gliadin** **(%)**	**Glutenin** **(%)**
N_0_	65.98 ± 1.70^**^	14.27 ± 1.16^**^	1.13 ± 0.06^**^	12.89 ± 0.39^**^	2.69 ± 0.35	1.48 ± 0.04^*^	2.59 ± 0.19^**^	4.17 ± 0.12^*^
N_ck_	72.18 ± 2.83	19.83 ± 1.26	1.42 ± 0.03	15.83 ± 0.57	2.80 ± 0.15	1.67 ± 0.08	3.15 ± 0.02	4.86 ± 0.25

* *P < 0.05 significant difference,*

** *P < 0.01 extremely significant difference*.

**Figure 2:**
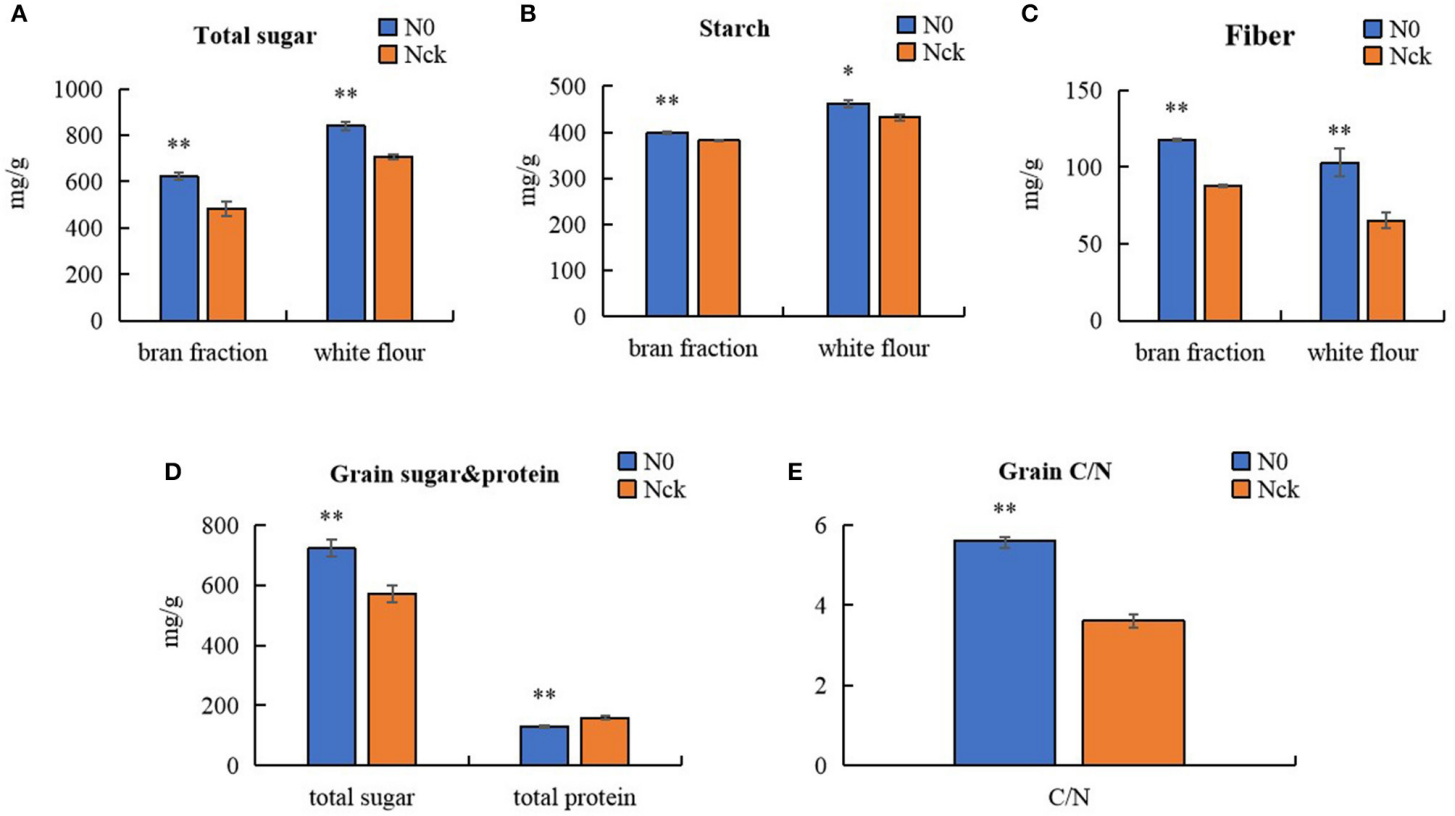
Content of total sugar, starch, and fiber in the bran fraction and white flour. **(A)** Total sugar in the bran fraction and white flour. **(B)** Starch in bran fraction and white flour. **(C)** Fiber content in the bran fraction and white flour. **(D)** Total sugar and total protein contents in matured grain. **(E)** Ratio of carbohydrate to protein, known as C/N. The blue columns represent the N0 treatment, orange columns represent the Nck treatment. *P < 0.05 significant difference, **P < 0.01 extremely significant difference.

Regarding protein quality, with the exception of albumin, which showed no significant difference, the contents of other protein types under N0 conditions were all lower than those of Nck. According to the differences in protein content, wheat was classified as high-gluten (>14%), medium-gluten (>12.5%), and low-gluten types (<12.5%) (GB/T 17320-2013, China). For Shiluan 02-1, the normal protein content is 15–16%, and was 15.83% under the normal N conditions in this study, which meets the standard of high-gluten wheat. However, the grain protein content decreased to 12.89% under N0 conditions, which only meets the standard of medium-gluten wheat. Therefore, the total grain protein content was reduced by 18.6% and was significantly affected by the low N conditions compared with the normal N conditions. In particular, N0-treatment had an extremely significant reduction in gliadin (17.8%), and a significant reduction in globulin (14.2%) and glutenin (14.2%). Therefore, N deficiency seriously affected the yield and quality of wheat and most indicators of leaf morphological and physiological functions, but increased grain weight and size. However, the contents of total sugar, starch, and fiber in N0 were all significantly higher than in Nck (Figure 2). The total sugar content in N0 grain was 26.5% higher than in Nck grain, which showed a similar tendency in the bran fraction and white flour. The total sugar of the bran fraction and white flour in N0 increased by 29.2 and 18.7%, respectively, compared that in Nck. The starch content of the bran fraction and white flour in N0 was 4.3 and 7% higher than that in Nck, while the fiber content of the bran fraction and white flour in N0 was 33.8 and 57.7% higher than that in Nck, respectively (Figure 2). Thus, the difference in total sugar was mainly determined by the content of fiber. In addition, the ratio of carbohydrate to protein, known as C/N, in N0 grain was extremely higher than that in Nck, which might together contribute to increasing the grain size and grain weight.

### Transcriptome Analysis of Wheat Grain Grown Under N Deficiency Conditions

**Figure 3:**
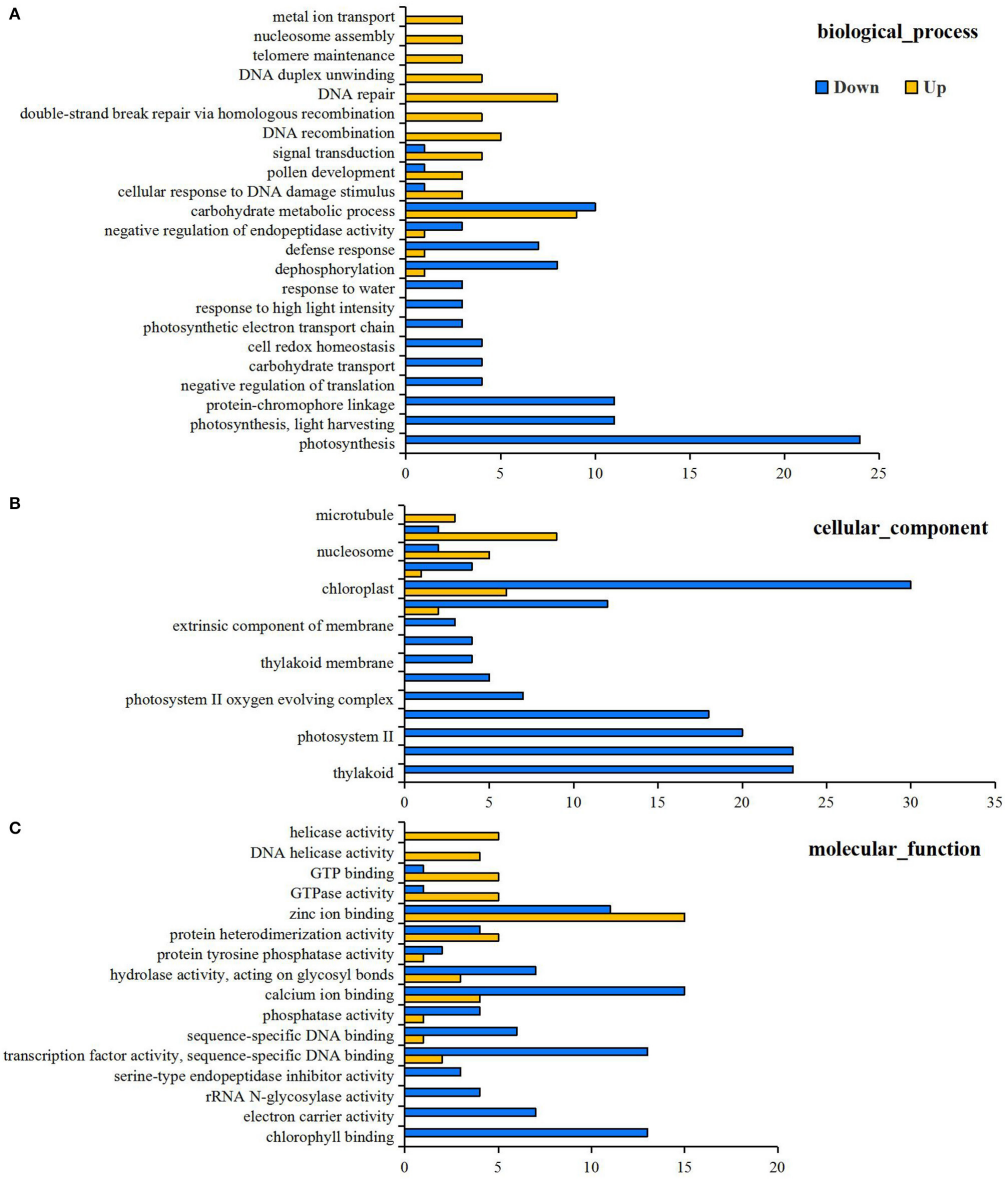
GO classifications of differentially expressed genes (DEGs) for mid-filling grain under N0 and Nck conditions, including three categories **(A)** biological process, **(B)** cellular component, and **(C)** molecular function. Yellow columns represent up-regulated items, and blue columns represent down-regulated items. The x-axis is the amount of DEGs involved related biological process, cellular component and molecular function.

RNA-Seq detected 77,642 genes with a suitable FPKM value, and the detailed information is listed in Supplementary Table 2. Principal component analysis analysis of gene expression for the N0 and Nck groups is shown in Supplementary Figure 2, which indicates that the reproducibility between the two groups was good and that sample selection was reasonable. A total of 761 genes were differentially expressed in the wheat grain samples under N deficiency conditions, and 363 genes were up-regulated and 398 genes were down-regulated (Supplementary Table 3). GO functional analysis annotated 462 GO terms based on 467 DEGs (Supplementary Table 4), and 54 significantly enriched GO terms were finally obtained [the limited criteria of FDR (false discovery rate) ≤ 5; No. of DEGs in one GO term ≥3], including 23 biological processes (BP), 15 cellular components (CC), and 16 molecular functions (MF). Among BP functions, the central DEGs down-regulated under N stress conditions were associated with photosynthesis, light harvesting, protein-chromophore linkage, carbohydrate metabolic process, cell redox homeostasis, and photosynthetic electron transport chain. In terms of CC, the down-regulated DEGs were mainly relevant to photosystem I (PSI), photosystem II (PSII), the chloroplast, thylakoid, plastid, membrane, and cell wall. Regarding the MF, the down-regulated DEGs were related to chlorophyll binding, electron carrier activity, rRNA N-glycosylase activity, serine-type endopeptidase inhibitor activity, and transcription factor (TF) activity (Figure 2). Meanwhile, the up-regulated DEGs were tightly associated with DNA repair, replication and recombination, activities relevant to chromosomes, nucleosomes, microtubules, metal ion transport, protein hetero-dimerization, zinc ion binding, and GTPase activity (Figure 3).

**Figure 4:**
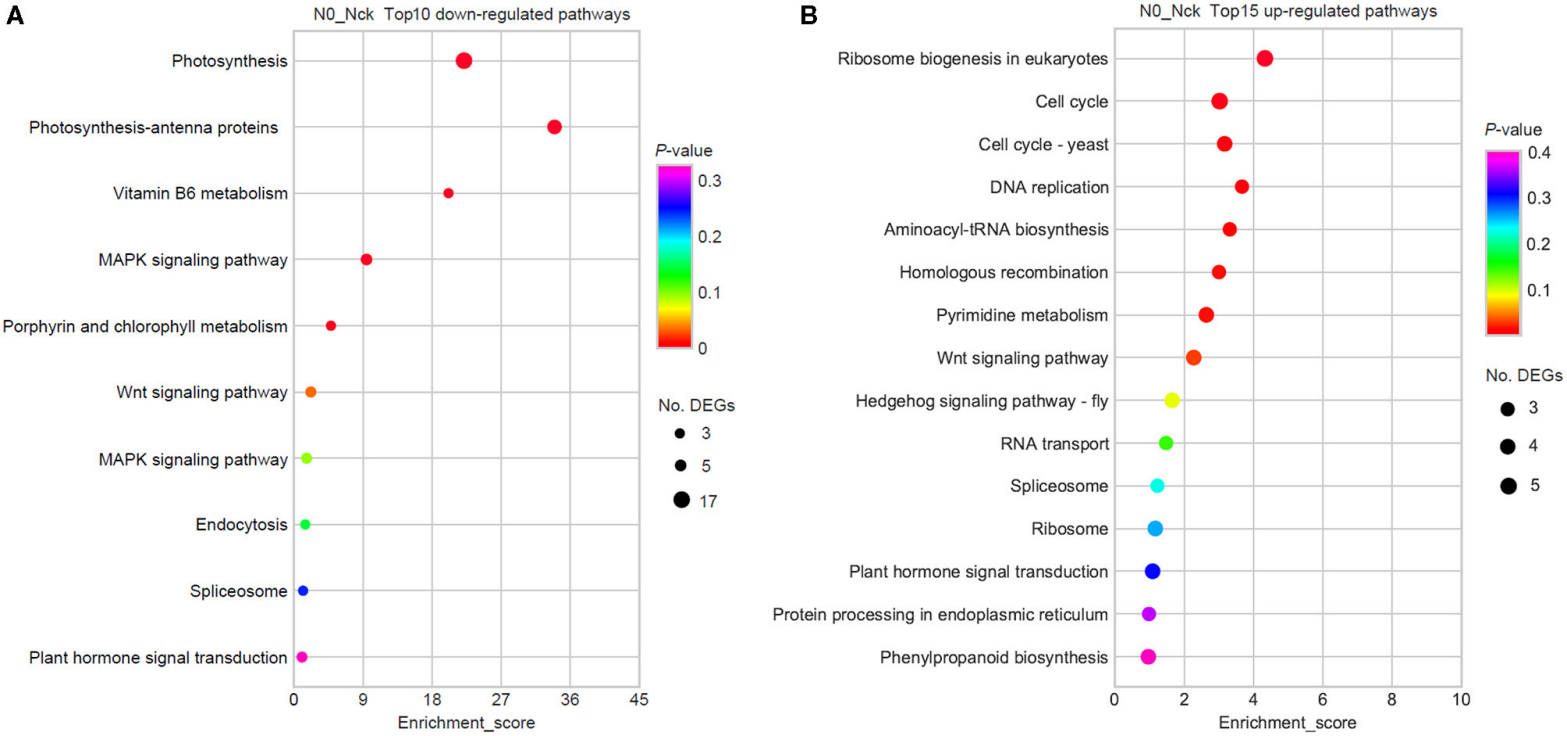
KEGG enrichment pathways by the DEGs. **(A)** Top 10 down-regulated pathways. **(B)** Top 15 up-regulated pathways. The x-axis is the enrichment score, which reflects the proportion of DEGs in a given pathway. The circle size indicates the number of DEGs in the pathway, and the larger circle is comprised of more DEGs. The bubble color changes from purple to blue, green, and red; and the smaller the enrichment *P*-value, the greater the statistical significance.

To further understand the active physiological processes of mid-filling grain under N deficiency conditions, the DEGs in various metabolic pathways were mapped to the KEGG database. It was found that there were 146 (out of 761) DEGs mapped to 105 KEGG pathways, 72 up-regulated genes were assigned to 81 KEGG pathways, and 74 down-regulated genes were assigned to 48 KEGG pathways (Supplementary Table 5). The top 10 lowly expressed and 15 highly expressed significant pathways were finally obtained (the limited criteria of *P*-value < 0.05; No. of DEGs in one KEGG pathway ≥3) (Figure 4). Photosynthesis, photosynthesis-antenna proteins, the MAPK and VEGF signaling pathways, and vitamin B6 metabolism were hindered because of N stress. However, the metabolic process regarding ribosome biogenesis in eukaryotes, the cell cycle, cell cycle-yeast, DNA replication, aminoacyl-tRNA biosynthesis, homologous recombination, and pyrimidine metabolism were increased to resist N stress.

### RNA-seq Validation by RT-qPCR

Thirteen genes were randomly selected to verify RNA-seq results. The down-regulated genes under N starvation conditions by RNA-seq (*P* < 0.05) included plastocyanin (*PETE)*, PS I reaction center subunit (*PS I-K*), chlorophyll a-b binding protein (*LHBC*), protochlorophyllide reductase (*PORA*), homolog gene *OsI_012078, calcineurin B-like protein 3* (*CBL3*), and two heat shock factor protein genes (*HSFB2A, HSP70*). The up-regulated genes included auxin (*PIN9*), histidine kinase (*mak2*), Wall-associated receptor kinase (*WAK5*), ribosome biogenesis in eukaryotes (*CDC48C*), and DNA activities (*SMC6B*) genes. Then, we took grains at the mid-filling stage with four consecutive years of N0 and Nck treatment as materials and analyzed the relative expression by RT-qPCR. The correlation coefficient of relative expression (log_2_FC) among the 13 genes with three biological replicates determined by RT-qPCR were extremely correlated (γ = 0.8238, *P* < 0.01) with those obtained from the RNA-seq (Supplementary Figure 4). Namely, the results confirmed the accuracy of RNA-seq in this study.

### Down-Regulated DEGs Associated With Photosynthesis by N Deficiency

**Figure 5:**
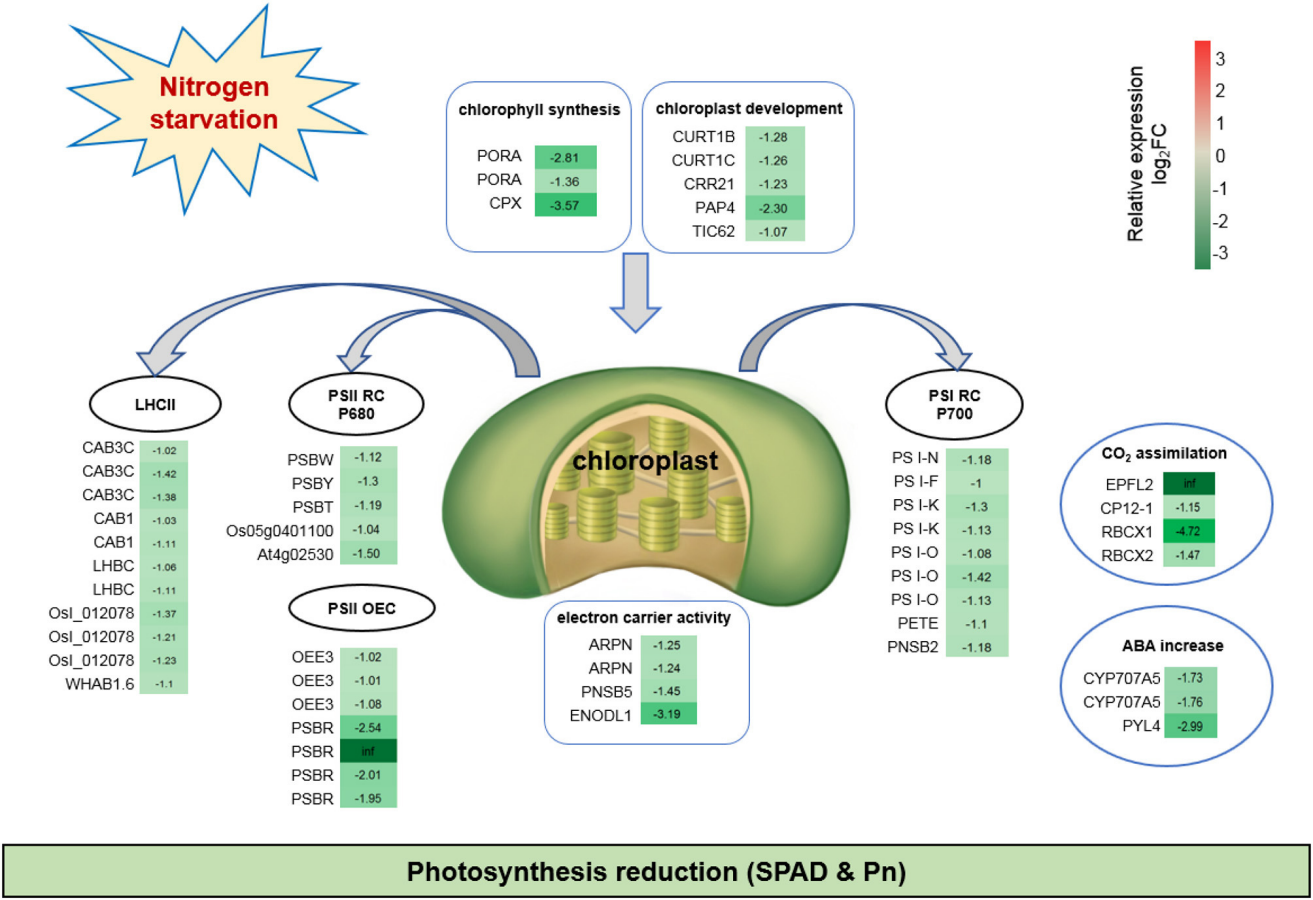
Fifty-one down-regulated DEGs suggesting inhibited photosynthesis under N starvation conditions. The relative expression levels as described by log_2_FC (FC is the fold change of RPKM means N0/Nck) are represented by a color gradient from low (green) to high (red). Under N-starvation conditions, the DEGs with log_2_FC > 0 represent up-regulated expression, log_2_FC < 0 represents down-regulated expression, and log_2_FC = 0 represents unchanged expression.

Photosynthesis was dramatically reduced upon adaptation to N-deficient conditions. Fifty-one DEGs were down-regulated after continuous low-N-stress, which indicated the inhibition of photosynthesis-related pathways, such as, chlorophyll synthesis, chloroplast development, PSI, PSII, light-harvesting chlorophyll (LHC) protein complex, electron carrier activity, CO_2_ assimilation, and stomatal closure (Figure 5). For example, the expressions of *PORA* and *CPX*, which are involved in porphyrin and chlorophyll metabolism, were notably lower in N0 compared to the Nck condition. Five DEGs hampered chloroplast development. Nine DEGs related to the PSI reaction center subunit protein decreased electron transport. Eleven DEGs associated with Chl a/b binding (CAB) proteins reduced light harvesting. During photosynthesis, water oxidation occurs in the oxygen-evolving complex (OEC). Three oxygen-evolving enhancer protein 3 (*OEE3*) and four PS II 10 kDa polypeptide (*PSBR*) genes associated with the OEC of PSII were down-regulated. Furthermore, N-deficiency also affected CO_2_ assimilation and the Calvin Cycle through *RBCX1* and *RBCX2*, which have been linked to the inhibition of stomatal guard cell development (Kolesiński et al., 2011), Rubisco activity, and chloroplast activity during dark periods (Marri et al., 2010).

### Amino Acid and Sugars Metabolism-Related DEGs by RNA-seq

**Figure 6:**
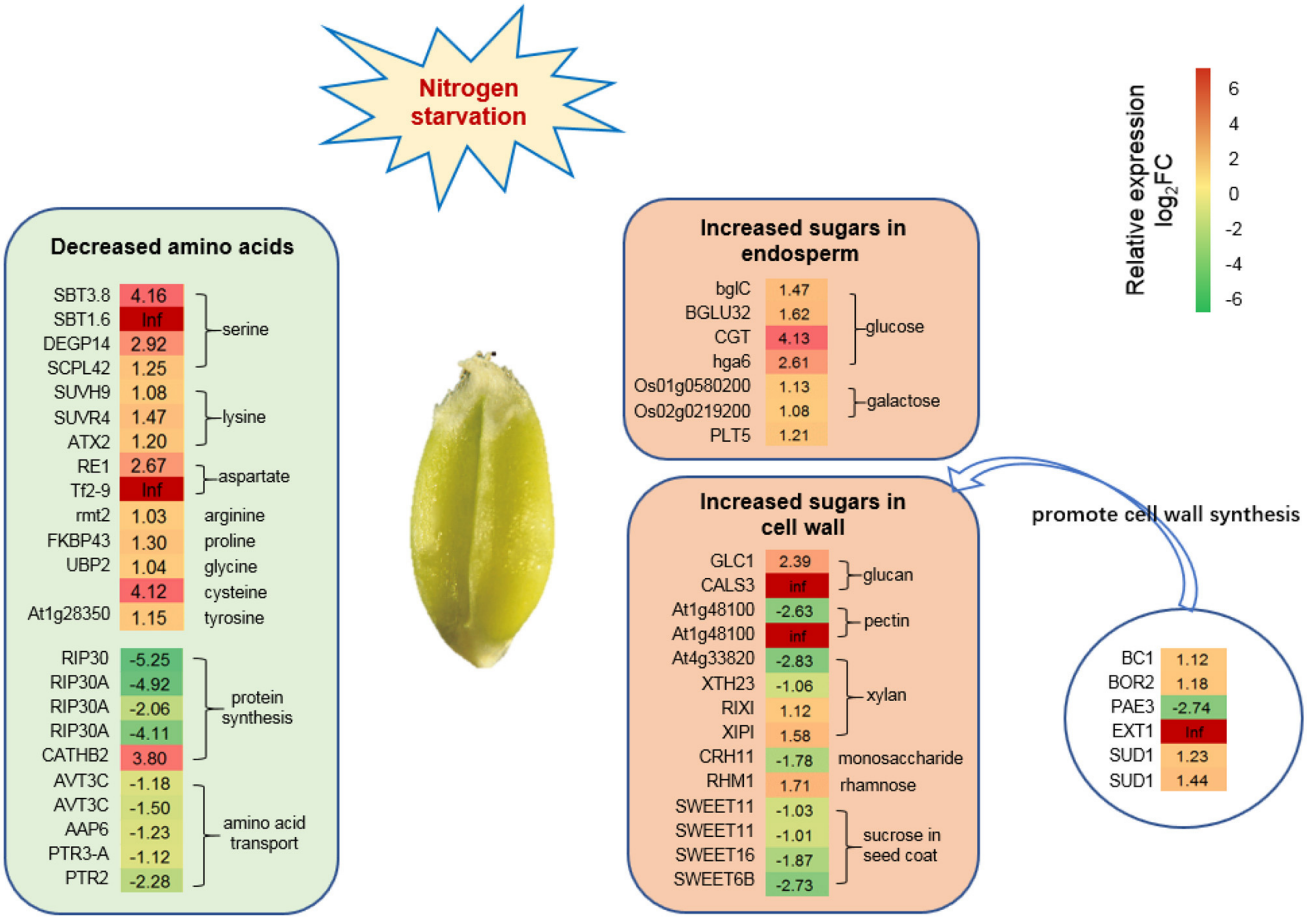
DEGs related to amino acid and sugar synthesis under N starvation conditions. Twenty-four DEGs associated with amino acid synthesis decreased the level of amino acids. Twenty-one DEGs associated with sugar synthesis increased the sugar level in the endosperm and episperm (mainly representing the cell wall). Six DEGs were significantly expressed that promote cell wall synthesis. The relative expression levels as described by log_2_FC (FC is the fold change of RPKM means N0/Nck) are represented by a color gradient from low (green) to high (red). Under N-starvation conditions, the DEGs with log_2_FC > 0 represent up-regulated expression, log_2_FC < 0 represents down-regulated expression, and log_2_FC = 0 represents unchanged expression.

The reduction of photosynthesis might affect amino acid and sugar metabolism in wheat grain grown under low N stress conditions. RNA-seq analysis showed that 24 DEGs might be associated with the reduction of amino acids synthesis or transport, such as serine (4), lysine (3), aspartic acid (2), arginine (1), proline (1), glycine (1), cysteine (1), tyrosine (1), and the inhibition of protein synthesis (5), and amino acid or peptide transport (5) (Figure 6). Twenty-one DEGs promoted the synthesis and transport of sugars, such as glucose (4), galactose (2), polyol (1), and sugars related to cell wall synthesis (14) (Figure 6).

### Up-Regulated DEGs Under N-Deficiency Conditions

**Figure 7:**
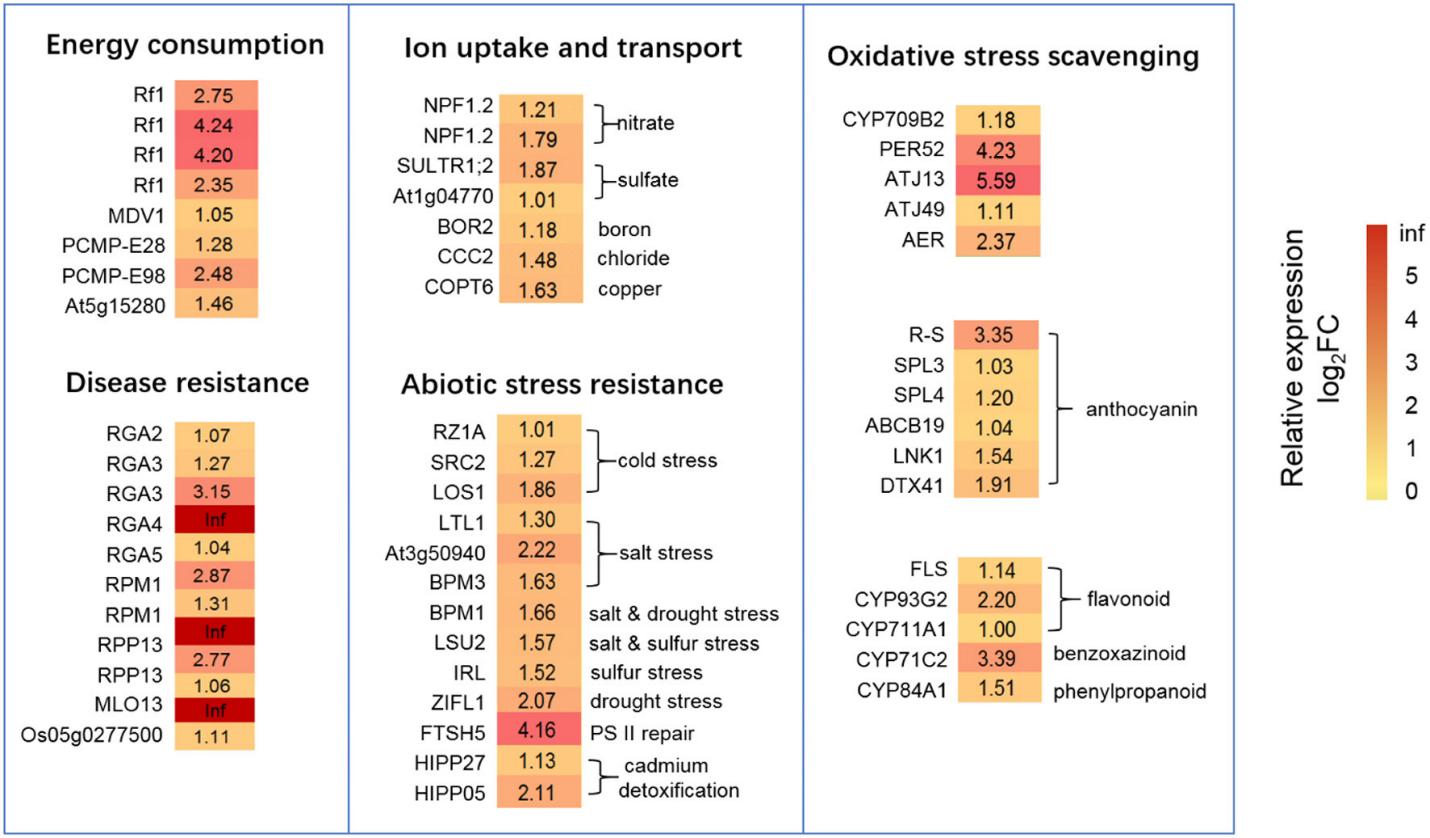
DEGs related to energy consumption, ion uptake and transport, disease resistance, abiotic stress resistance, and oxidative stress scavenging under N starvation conditions. The relative expression levels as described by log_2_FC (FC is the fold change of RPKM means N0/Nck) are represented by a color gradient from low (green) to high (red). Under N-starvation conditions, the DEGs with log_2_FC > 0 represent up-regulated expression, log_2_FC < 0 represents down-regulated expression, and log_2_FC = 0 represents unchanged expression.

In order to resist N starvation, most of the up-regulated DEGs were related to energy consumption, ion uptake and transport, disease, abiotic stress resistance, and oxidative stress scavenging. Seventy-one DEGs were increased, mainly involving the structural constituents of ribosomes (4), ribosome biogenesis in eukaryotes (8), RNA transport, splicing, and modification (18), DNA replication, DNA recombination, DNA repair, chromatin stabilization (22), the cell cycle and division (16), and cell proliferation (3) (Supplementary Table 6). However, energy consumption was also increased based on the above changes, as eight mitochondrial-associated genes were up-regulated, especially four *Rf1* genes increased by 2.35- to 4.26-fold (log_2_FC) with N0 treatment (Figure 7). Ten DEGs were related to iron uptake and transport, of which seven DEGs were up-regulated (log_2_FC = 1.01–1.87) to promote nutrient iron assimilation and transport, including low-affinity nitrate, sulfate, boron, chloride, and copper under N-deficient conditions (Figure 7; Supplementary Table 7). Seventeen DEGs related to disease resistance ability were identified, and 71% of these were up-regulated (Figure 7; Supplementary Table 7). Eighteen DEGs related to abiotic stress resistance were significantly expressed, of which 72% were up-regulated (log_2_FC = 1.01–4.16) to resist N starvation, mainly including resistance to cold stress (3 DEGs), salt stress (5 DEGs), sulfur-deficiency stress (2 DEGs), drought stress (2 DEGs), cadmium toxication (2 DEGs), and photosystem repair (1 DEG) (Figure 7; Supplementary Table 7). In addition, 13 DEGs were up-regulated to elevate antioxidant defense (Figure 7). Two chaperone protein genes (*ATJ13, ATJ49*), a peroxidases gene (*PER52*), and an NADPH-dependent oxidoreductase gene (*AER*) were contributing to the removal of H_2_O_2_ and toxic reductants. Three TFs (*R-S, SPL3, and SPL4*) and three genes (*ABCB19, LNK1*, and *DTX41*) promoted the accumulation of anthocyanidin. Moreover, the higher expression of *FLS* and *CYPs* family genes participated in flavonoid, benzoxazinoid, or phenylpropanoid biosynthesis to improve oxidation resistance ability.

### Metabolomics Analysis

A total of 280 detectable metabolites and their relative content levels are shown in a data matrix (Supplementary Table 8). The data show that 77 differentially accumulated metabolites were detected under N deficient conditions, namely, sugars (17), organic acids (14), amino acids (12), fatty acids (8), amines (8), lipidol (5), nucleic acids (3), and others (10) (Table 3). The clustering heatmap of content levels for differential metabolites with N0 and Nck is shown in Supplementary Figure 3. It can be seen that the repeatability of six experiment groups and six control groups was excellent and presented a significant degree of separation of N0 and Nck. In response to N deficiency, 61% of the metabolites were down-regulated in wheat grain, including all nucleic acids, 71% of organic acids, 75% of amino acids, 75% of fatty acids, 80% of the lipidol, and 88% of amines. In addition, 39% of the metabolites were up-regulated with N0, which mainly included 94% of the sugars. It is worth noting that the contents of arachidonic acid and hexadecane were increased under N stress conditions, 83,937 and 2,113,971-fold compared with normal N fertilizer application, respectively.

**Table 3:** Seventy-seven metabolites identified in wheat grain with N0 and Nck treatments.

**Group**	**Metabolites**	**VIP**	**FC N0/Nck**	**Regulation**
**Sugars**	3,6-Anhydro-D-galactose	1.42	0.64	Down
	Tagatose	1.55	1.20	Up
	Myo-inositol	1.62	1.30	Up
	Ribose	1.65	1.31	Up
	Galactinol	1.54	1.32	Up
	Gluconic acid	1.88	1.33	Up
	N-Acetyl-beta-D-mannosamine	1.65	1.36	Up
	Mannitol	1.58	1.38	Up
	1,5-Anhydroglucitol	1.33	1.39	Up
	D-Talose	1.55	1.50	Up
	Lyxose	1.55	1.55	Up
	Sophorose	1.51	1.61	Up
	Trehalose	1.38	1.76	Up
	Turanose	1.49	1.90	Up
	D-Galacturonate	1.39	2.46	Up
	Isopropyl-beta-D-thiogalactopyranoside	1.57	1.58	Up
	Leucrose	1.09	2.01	Up
**Organic acids**	Toluenesulfonic acid	1.63	0.36	Down
	Benzoic acid	1.83	0.47	Down
	Pipecolinic acid	1.48	0.50	Down
	Citric acid	1.63	0.60	Down
	Tartronic acid	1.22	0.66	Down
	2-Furoic acid	1.95	0.68	Down
	Glucosaminic acid	1.43	0.69	Down
	Oxalic acid	1.45	0.77	Down
	5-Methoxyindole-3-acetic acid	1.64	0.52	Down
	5-Hydroxyindole-2-carboxylic acid	1.61	0.74	Down
	GABA	1.56	1.21	Up
	Gly-pro	1.65	1.22	Up
	4-Hydroxybutyrate	1.46	1.33	Up
	Maleic acid	1.65	4.81	Up
**Amino acids**	Norvaline	1.01	0.42	Down
	Ornithine	1.26	0.58	Down
	L-Cysteine	1.51	0.63	Down
	Aspartic acid	1.57	0.66	Down
	Cysteinylglycine	1.31	0.80	Down
	Carnitine	1.55	0.81	Down
	N-Alpha-acetyl-L-ornithine	1.47	0.82	Down
	N-Methyl-DL-alanine	1.48	0.82	Down
	Tyrosine	1.48	0.83	Down
	Beta-alanine	1.51	1.21	Up
	Beta-glutamate	1.41	1.40	Up
	5-Hydroxytryptophan	1.83	3.70	Up
**Fatty acids**	Trans, trans-muconic acid	1.10	0.48	Down
	Itaconic acid	1.69	0.60	Down
	3-Hydroxybutyric acid	1.37	0.74	Down
	5-Aminovaleric acid lactam	1.42	0.77	Down
	Methyl octanoate	1.62	0.79	Down
	5-Aminovaleric acid	1.41	0.83	Down
	Citraconic acid degr1	1.28	6.15	Up
	Arachidonic acid	1.92	83,936.97	Up
**Amine**	DL-Anabasine	1.69	0.14	Down
	Maleimide	1.41	0.58	Down
	Melatonin	1.21	0.67	Down
	2-Aminoethanethiol	1.60	0.75	Down
	Putrescine	1.38	0.78	Down
	5-Methoxytryptamine	1.34	0.84	Down
	Oxamide	1.60	0.61	Down
	Nicotianamine	1.70	2.21	Up
**Lipidol**	Shikimic acid	1.56	0.76	Down
	Glycerol	1.54	0.80	Down
	Farnesol	1.38	0.82	Down
	Cumic acid	1.43	0.82	Down
	Benzyl alcohol	1.64	3.11	Up
**Nucleic acid**	2'-Deoxycytidine 5'-triphosphate degr prod	1.71	0.58	Down
	5,6-Dihydrouracil	1.49	0.73	Down
	Thymine	1.83	0.60	Down
**Others**	4-Vinylphenol dimer	1.54	0.80	Down
	2'-Hydroxyacetophenone	1.42	0.81	Down
	p-Benzoquinone	1.54	0.81	Down
	Dibenzofuran	1.45	0.82	Down
	1-Methylhydantoin	1.35	0.82	Down
	Phosphate	1.24	0.51	Down
	D-Fructose 1,6-bisphosphate	1.83	0.49	Down
	1,3-Cyclohexanedione	1.43	3.96	Up
	5,6-Dimethylbenzimidazole	1.58	4.50	Up
	Hexadecane	1.63	2,113,971.43	Up

**Figure 8:**
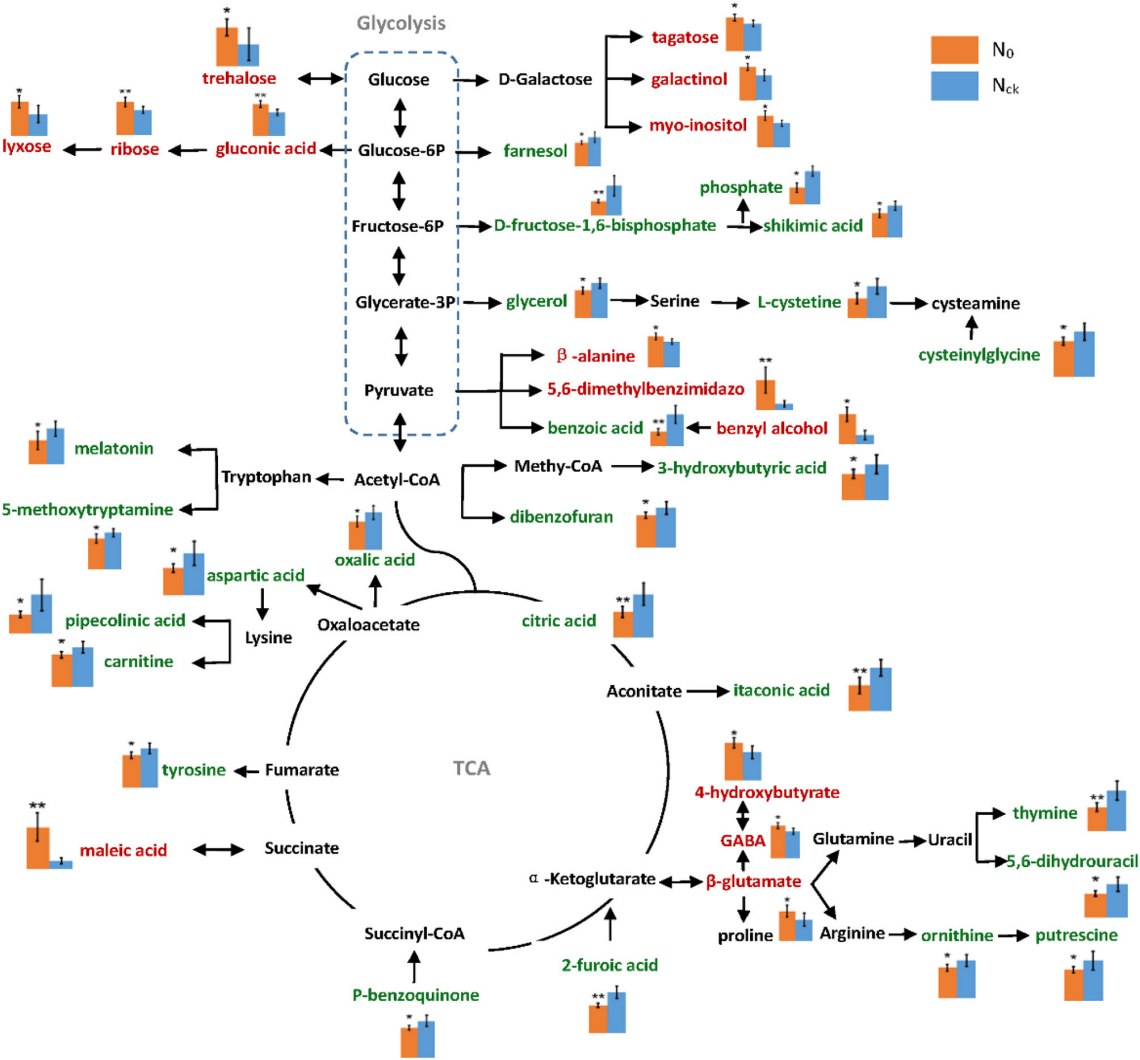
Metabolic pathway network of differentially accumulated metabolites under N0 and Nck conditions. The relative content levels of metabolites are shown in column charts: orange color column represents N0, and blue color represents Nck. The words in green color represent down-regulated metabolites, and those in red color represent up-regulated metabolites. The errors are shown in all bar graphs, and a significant difference between N0 and Nck in each metabolite is also evaluated, *P < 0.05 significant difference, **P < 0.01 extremely significant difference.

A metabolic network diagram based on 39 DAMs of wheat grain in the mid-filling stage is shown in Figure 8. From this network, it is apparent that the down-regulated metabolites (64%) were mainly involved in N metabolism, as indicated by the significant decrease in amino acid content of ornithine, L-cysteine, cysteinylglycine, aspartic acid, and tyrosine by 17–42%, which resulted in a 18.6% reduction in the crude protein content of mature grains. This is likely due to the significant reduction of many intermediate metabolites of the tricarboxylic acid cycle (TAC) under N deficiency conditions. The down-regulated metabolites included pipecolinic acid and carnitine in aspartic acid as well as lysine metabolism, maleimide and 5-methoxytryptamine in tryptophan metabolism, and putrescine in ornithine and arginine pathways. In order to resist N deficiency, sugar-related metabolites increased, particularly those of the pentose phosphate pathway (PPP), such as trehalose, lyxose, gluconic acid, and ribose. This is likely because of the impediment of glycolysis under N0.

## Discussion

### Photosynthesis Was Inhibited Under N-Deficiency

Nitrogen deficiency is a significant limiting factor of growth, grain yield, and grain quality of wheat, a crop that has a high N fertilizer demand (Salvagiotti and Miralles, 2008; Masclaux-Daubresse et al., 2010; Liu et al., 2020b). Chronic nitrogen starvation negatively affected plant height, tillering, flag leaf area, spike and seed traits, and grain total N content in durum wheat (Curci et al., 2017). Here, we also found that low-N treatment significantly decreased plant height, leaf length and width, grains/spike, and spikes/hm^2^, and resulted in a decrease in grain yield and protein content by 19.3 and 18.6%, respectively, under N0 conditions compared with the Nck conditions. Chloroplasts are vital organelles of photosynthetic cells in plants (Kirchhoff, 2013), and ~75% of N is allocated to chloroplasts to synthesize the photosynthetic apparatus, including thylakoid membranes and related enzymes. Therefore, insufficient N fertilizer influences chloroplast structure (Bondada and Syvertsen, 2005) and decreases chlorophyll content, leading to yellowing leaf (Diaz et al., 2006) and ultimately impeding photosynthesis (Antal et al., 2010; Wei et al., 2016). We found that photosynthesis was the most heavily influenced pathway by N-deficiency through GO and KEGG analyses. *PORA* homologs, associated with *PORA*, were down-regulated 1.36- to 2.81-fold under N0 conditions (Figure 5), and a similar result was also found in Arabidopsis, resulting in a reduction of chlorophyll levels by ~30% under tested N limiting conditions (Bi et al., 2007). From the phenotypic observations of the authors, the SPAD value and Pn of flag leaf decreased by 32 and 15.2%, respectively, consistent with the transcriptome results. Generally, the predominant down-regulation of genes related to photosynthesis and light harvesting has been often observed in wheat grain during the early filling stage (Sultana et al., 2020). Here, we also observed a similar phenomenon in medium-filling wheat grain in response to N-deficiency, which was a continuous stress adaption for wheat grain.

In a complete photosynthetic system, the PSI and PSII complexes harvest and transfer light to the photosynthetic reaction center known as P680 for PSII and P700 for PSI to initiate electron transport and energy transfer (Ruban, 2014). Here we found the DEGs relative to PSI reaction center complexes included *PSI-N, PSI-F, PSI-K* (2), *PSI-O* (3), *PSI-P, PETE*, and *PNSB2*, which showed a reduced response to N-deficiency. These DEGs was previously reported that linked with plastocyanin docking to the PSI complex and carried out effective electron transport (Jensen et al., 2004; Khrouchtchova et al., 2005). PET family genes were found to be down-regulated in wheat shoot and root under N-starvation conditions to reduce photosynthesis (Xin et al., 2020). Photosystem II reaction center complexes such as *PSBR* protein were down-regulated in wheat seedling (Wang et al., 2019) and wheat grain under N stress conditions (Sultana et al., 2020). Here, we also found four down-regulated PSBR genes inhibited photosynthesis (Figure 5), which was consistent with the previous results. Each photosystem comprises a core complex and a peripheral antenna system, light-harvesting complex I (LHCI) for PSI, and light-harvesting complex II (LHCII) for PSII, respectively. Light-harvesting complex II combines ~50% of photosynthetic pigments and nearly 1/3 of chloroplast membrane proteins, and is a major photo-capture and transfer complex in PSII (Standfuss et al., 2005). The CAB proteins are a highly conserved family of nuclear-encoded proteins that belong to the LHC protein family (Dolganov et al., 1995). Eleven *CAB* genes belonging to the LHCII type were all down-regulated including *CAB-1* (2), *CAB-3C* (3), the homolog gene *LHBC* (2), *OsI_012078* (3), and *WHAB1.6* (1) (Figure 5), which was also the most down-regulated gene in wheat leaves and grain during and after anthesis under N-deficiency conditions (Sultana et al., 2020). Abscisic acid (ABA) plays a key role in promoting stomatal closure (Cai et al., 2017). Here, the down-regulation of *CYP707A5* (2) and *PYL4* likely restrained ABA degradation and also resulted in stomatal closure and the reduction of photosynthesis (Figure 5), which was an adaptation to low-Nin wheat. Similar mechanisms were demonstrated in *Arabidopsis* guard-cells in response to drought (Virlouvet and Fromm, 2015).

### Adaptive Mechanism of Amino Acids and Sugars Metabolism Under N-Deficiency Conditions

Amino acids are the major form in which N is remobilized from leaves to the grain during grain filling (Howarth et al., 2008). An insufficient N supply in leaves resulted in a reduction of protein and amino acids in wheat grain, especially leucine and phenylalanine (Zhang et al., 2016). Here, we found cysteine and aspartate were all decreased by RNA-seq and metabolic analyses, which may be the main reason for the reduction of gluten content. However, some amino acids or organic acids such as proline, glutamate, and γ-aminobutyric acid (GABA), are also synthesized in large amounts when plants experience stress, i.e., wheat and barley in cold and drought stress, and a higher accumulation of glutamate will induce more substantial generation of GABA (Mazzucotelli et al., 2006; Vergara-Diaz et al., 2020). In this study, the metabolomic analysis demonstrated that the TCA cycle was inhibited by insufficient N-supply in the whole growth stage, leading to a large accumulation of intermediate products such as β-glutamate and GABA (Figure 8). Moreover, RNA-seq data also indicated the glutamate synthesis genes, such as *GATB*, the homologous gene to *At1g74260*, and *GLR3.4* genes were expressed at high levels (Supplementary Table 3), which might enhance the accumulation of glutamate. The top up-regulated genes found in wheat grain at 10 DAP were glutamate dehydrogenase and glutamine synthase (Sultana et al., 2020), which was a tolerance mechanism to low N conditions, in agreement with the results.

Interestingly, although amino acid levels in grain decreased, the TGW increased under N-deficiency conditions. The mature wheat grain comprises three major components, about 60–70% starch, 12–18% proteins, and 12–16% fiber (Andersson et al., 2013), which, together, account for ~90% of the dry weight (Shewry et al., 2013). In the experiment, the protein content decreased by 18.6% with N0 treatment. However, the TGW of Nck increased by 12.5% under N0 conditions, which demonstrated that the 31.1% difference in grain weight was mainly caused by the difference in starch and fiber content. The major components of fiber and starch are all polysaccharides, thus an increase in TGW is linked to an increase in sugar accumulation. Under field conditions, the No. grains/spike and No. spikes/hm^2^ were reduced by 25.6 and 3.7%, respectively, with N0 treatment compared with Nck treatment, which caused fewer grains per unit area and provided more N supply for each grain. Together, these factors might be responsible for the higher C:N ratio in the N0 wheat plants compared with the Nck plants, which thereby promoted sugar synthesis and transport in grain, and ultimately resulted in increased total sugar content and C/N ratio of N0 grain by 26.5 and 55.3%, respectively, compared with that of Nck grain (Figure 2).

Here, we found the difference in total sugar of N0 and Nck was mainly due to the fiber content difference in both bran fraction and white flour, including the aleurone layer and endosperm. Wheat fiber mainly comprises non-starch polysaccharides derived from the cell walls (Gebruers et al., 2010), which contain about 70% arabinoxylan and 20% β-glucan (Mares and Stone, 1973). RNA-seq results have revealed that six significantly expressed genes induced an increase in xylan and glucan, another eight homologous genes also suggest increased galactosan, sucrose, and monosaccharide contents, as well as six genes that promoted cell wall synthesis. Together, these changes promoted sugar accumulation in the cell wall and increased fiber content in matured grain under N-deficiency conditions. Moreover, the weight proportion of fiber content in total sugars of the N0 bran fraction and white flour was 18.9 and 12.2%, respectively, which was higher than that of the Nck bran fraction (18.21%) and white flour (9.21%) (Figure 2); these data indicated that fiber content in endosperm was significantly increased under N0 conditions. Previous studies have stated that the extensibility of the cell wall can be increased by the overexpression of expansin genes in wheat roots to increase N absorption efficiency under low-N stress conditions (Xin et al., 2020). The cell wall was shown to thicken and had a higher galactose content following N stress in *Arabidopsis* (Chrost et al., 2007; Pandey et al., 2017). As a consequence, the increase in fiber components such as xylan and glucan in endosperm might be a key reason for higher TGW under N-deficiency conditions.

Starch generally accounts for about 80% of the dry weight of starchy endosperm, which is largely responsible for the increase in grain size and weight (Shewry et al., 2013). However, only 55–64% of starch in total sugar was determined in bran and white flour, which indicated that soluble sugars were not transformed to starch under N-deficiency conditions. RNA-seq and metabolomic analyses also identified that soluble sugars such as galactose and glucose were increased with N0 treatment, which likely occurred through the up-regulation of four metabolites (3,6-anhydro-D-galactose, galactinol, galacturonic acid, and isopropyl-β-D-thiogalactopyranoside) and two gene homologs (*Os01g0580200, Os02g0219200*) related to galactose synthesis, and up-regulation of two metabolites (gluconic acid and 1,5-anhydroglucitol) and four genes (*bglC, BGLU32, CGT*, and *hga6*) related to glucose synthesis (Figure 6; Table 3).

The PPP in plants accounts for more than 50% of sugar metabolism and is responsive to stresses. Recent studies have reported that the PPP plays an important role in tolerating nitrate stress (Lin et al., 2020) and cold acclimation responses (Sarkar and Bhowmik, 2009). In the PPP, glucose is directly oxidized and decomposed independently of glycolysis and the TAC. According to the data, the PPP was altered by the up-regulation of trehalose, lyxose, ribose, gluconic acid, tagatose, galactinol, and myo-inositol, indicating that glycolysis was inhibited to a certain extent to protect wheat under N deficiency conditions (Figure 8). Sugars such as sucrose and inositol protect cell membranes during cold stress (Janská et al., 2010). Therefore, the increase in sugars is not only likely a cause of the increase in TGW but also a response mechanism to N stress.

### Resistance Mechanisms of Wheat Grain to N-Deficiency Stress

Wheat has developed many mechanisms to facilitate tolerance to N deficiency, such as enhancing nucleotide metabolism, nitrogen and sulfate transport, and steroid biosynthesis (Guo et al., 2014; Sultana et al., 2020; Xin et al., 2020). Here, the up-regulation of *SMC6B, BRCA1, BARD1*, and FAS2 likely facilitates the repair of double-stranded DNA breaks *via* homologous recombination. Boyko et al. (2010) also found that *Arabidopsis* plants confirmed a higher frequency of homologous recombination in response to abiotic stress under the exposure of stressors such as heat, cold, salt, flood, and ultraviolet radiation, which is consistent with the findings. Histone-related genes such as *TH123, H2B.2*, and *hh4* play a central role in transcriptional regulation, upon which upregulation promotes chromosome stability, DNA repair, and replication, as well as regulated chromatin remodeling under low-N conditions. Moreover, evidence has pointed to chromatin remodeling as a key mechanism in promoting plant stress responses (Ding et al., 2012). Sultana et al. (2020) found that DEGs related to nucleotide metabolism of wheat leaf and grain were positively up-regulated under N-starvation conditions at anthesis and 10 days after anthesis. This study showed similar results at the grain-filling stage, which reflected a similar stress resistance mechanism to N-deficiency at different growth stages.

Wheat can improve iron uptake and transportability to resist N-deficiency, such as by up-regulating high-affinity transporters of the *NRT1/PTR family protein 2.2* (Sultana et al., 2020). Here, we observed low-affinity nitrate transporters (*NPF1.2*) involved in xylem-to-phloem transport of nitrate into developing leaves (Figure 7) (Hsu and Tsay, 2013). Lower CTK levels might promote the transport of high-affinity ${\text{NO}}_{3}^{-}$ (Ruffel et al., 2011). *CKX3* encodes a cytokinin dehydrogenase and functions in catalyzing the degradation of CTK (Werner et al., 2003). *CKX3* up-regulation (Supplementary Table 9) will reduce CTK levels and enhance the assimilation of high-affinity ${\text{NO}}_{3}^{-}$ and N-deficiency tolerance, consistent with wild barley under N stress conditions (Quan et al., 2016). In addition, a sulfate transporter was reported to be more highly expressed in wheat leaf and grain during the anthesis period under N-stress conditions (Sultana et al., 2020). Here, the high-affinity H^+^/sulfate cotransporter (*SULTR1;2*) and sulfur deficiency-induced two homologous genes of *At1g04770* were up-regulated, which likely promoted sulfate assimilation for grain under N-deficiency conditions (Rouached et al., 2008; Howarth et al., 2009) (Figure 7), Disease resistance proteins guard the plant against pathogens (Cesari et al., 2013). We found that the expression of nine wheat homologs encoding disease resistance proteins was increased under LN stress conditions, including two *RGA2* homologs, *RGA3, RGA4, RGA5*, two *RPM1* homologs, and two *RPP13* genes (Figure 7). *FTSH5* is involved in thylakoid formation (Järvi et al., 2016) and in the removal of damaged protein in PSII, which contributes to the prevention of cell death under high-intensity light conditions (Kato et al., 2009), which likely promoted the repair of PS II under N stress conditions (Figure 7). Cadmium is a toxic element to plants. The two heavy-metal-binding protein genes, *HIPP05* and *HIPP27*, involved in cadmium detoxification (Abreu-Neto et al., 2013) were up-regulated upon N deficiency (Figure 7).

Under N starvation conditions, the electron transport system is hindered, which induces a massive accumulation of reactive oxygen species (ROS) (Shin et al., 2005; Lin et al., 2020). In order to resist oxidative stress, crops such as rice (Lian et al., 2006) and wheat (Wang et al., 2019) have developed antioxidant defense systems to detoxify ROS. Cytochrome P450s (CYPs) play a vital role in ROS detoxification (Mizutani and Ohta, 2010). Currently, we found that five CYPs DEGs were up-regulated (log_2_FC = 1–3.39) (Figure 7), which is in agreement with findings in barley at the seedling stage (Quan et al., 2016) and durum wheat at the late milk developmental stage (Curci et al., 2017). In Arabidopsis, the biosynthesis and accumulation of phenylpropanoids (including anthocyanin) were increased by N deficiency (Bollivar, 2006; Diaz et al., 2006). Here, the phenylpropanoid biosynthesis pathway (Figure 4) and DEGs related to anthocyanin and phenylpropanoid bisosynthesis (Figure 7) were together increased in wheat grain under N-deficiency condition, it should be considered as a N-stress resistant response, which was also observed in the wheat leaf (Sultana et al., 2020).

It has been proposed that some phytohormones, TFs, and protein kinases (PKs) coordinate the demand and acquisition of N (Argueso et al., 2009; Curci et al., 2017). In this study, the DEGs associated with hormones, TFs, and PKs under N-deficiency conditions are shown in Supplementary Table 9. Transcription factors, as important natural regulators for gene expression, seem to play a role in response to abiotic stress (Liu et al., 2014). Seven TFs related to ethylene synthesis were down-regulated, which may slow down leaf senescence and grain maturation process. Moreover, aminocyclopropane-1-carboxylate (ACC) oxidase (*ACO*) is a rate-limiting enzyme in ethylene synthesis and was previously shown to be down- regulated under low-N stress conditions (Quan et al., 2016). The up-regulation of *ARF11* and *PIN* would promote auxin accumulation and enhance grain-filling in the midterm of N0 treatment, consistent with the sugar increase in grain discussed above. Brassinosteroids (BRs) are a type of steroid that promote photosynthetic activity (Sakamoto et al., 2006) and are also reported to be involved in enhancing wheat N-deficiency tolerance and rice drought tolerance (Hayes, 2019). Here we found that *pif1*, a typical bHLH TF that negatively regulates BRs signaling (Wang et al., 2009), was down-regulated under low-N stress conditions. *FAR1* (far-red impaired response 1) is a TF in the phytochrome A (phA) signaling pathway that has been implicated in improving drought resistance in Arabidopsis (Tang et al., 2013) and regulates starch synthesis metabolism (Casal, 2000). Here, we found two *FRS5* genes were up-regulated, which increased the tolerance to low N-stress. TIFY is a plant-specific TF, and the over-expression of *TIFY10* in wild bean increased the ability to cope with salt stress (Zhu et al., 2011). Here, we found that two *TIFY10c* genes were up-regulated by 1.59- and 2.38-fold (log_2_FC) with N0 treatment, respectively. Leucine-rich repeat receptor-like kinases (*LRRs*), serine threonine-protein kinase (*STKs*), and wall-associated kinases (*WAKs*) are key PKs that participate in cell signal transduction response to abiotic stress (Rudrabhatla and Rajasekharan, 2002; Molle and Kremer, 2010). Here, 11 genes encoding *LRRs* (3), *STKs* (4), and *WAKs* (4) were upregulated, which may be involved in plant defense signaling during N stress. *WAKs* are signaling receptors at the cell surface and may contribute to cell wall synthesis. *AHK1* is a histidine kinase and positive regulator of drought and salt stress responses (Tran et al., 2007), and the up-regulation of histidine kinase genes that we observed (*AHK1* and *mak2*) might be involved in a resistance mechanism to N stress.

## Conclusion

This study carried out a complete assessment of the impact on distinct N regimes applied at the seedling stage with continuous application on filling and maturing wheat grains through phenotypic, transcriptomic, and metabolomic measurements. The results showed that photosynthesis was dramatically decreased during grain filling under N-deficiency conditions throughout the entire growth period. The higher C/N ratio under N-deficient conditions decreased grain protein content, but enhanced sugar and starch synthesis per grain to increase the TGW, which we posit as due to increased cell wall synthesis and fiber content in the endosperm. However, only one N-application rate was selected for transcription and metabolites analysis, which might be inadequate in explaining the molecular mechanisms of different N application regimens in field production. Therefore, additional treatments should be considered for future study.

## Data Availability Statement

The original contributions presented in the study are publicly available. This data can be found here: NCBI repository, accession: PRJNA707202.

## Author Contributions

YW contributed to the experimental design, yield and quality indicator measurements, data analyses, and original draft preparation. DW conducted data analyses, sequence BLASTs, and gene annotation, as well as measured the content of starch and fiber. GZ and XC conceived the study, carried out field management, and revised the manuscript. ZT, ZG, and YY helped with sample preparation and Pn and SPAD flag leaf measurements. All authors contributed to the article and approved the submitted version.

## Conflict of Interest

The authors declare that the research was conducted in the absence of any commercial or financial relationships that could be construed as a potential conflict of interest.

## Publisher's Note

All claims expressed in this article are solely those of the authors and do not necessarily represent those of their affiliated organizations, or those of the publisher, the editors and the reviewers. Any product that may be evaluated in this article, or claim that may be made by its manufacturer, is not guaranteed or endorsed by the publisher.
