# Supplementary material for: Impacts of Nitrogen Deficiency on Wheat (Triticum aestivum L.) Grain During the Medium Filling Stage: Transcriptomic and Metabolomic Comparisons
Source: Front Plant Sci. 2021 Aug 4;12:674433. doi: 10.3389/fpls.2021.674433 (PMC8371442; doi:10.3389/fpls.2021.674433)
Supplement: Supplementary file 1 [file Data_Sheet_1.PDF]

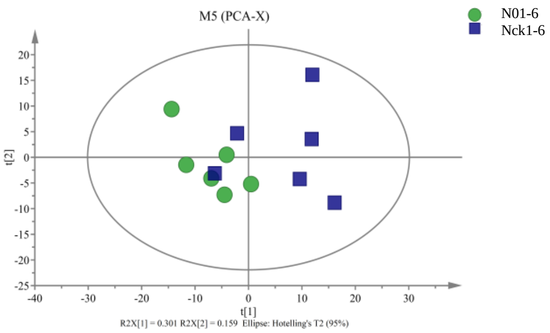

(a)

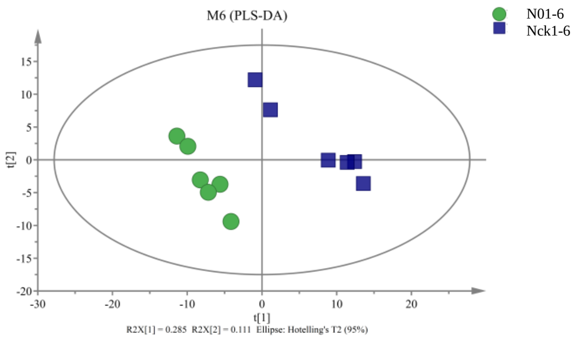

(b)

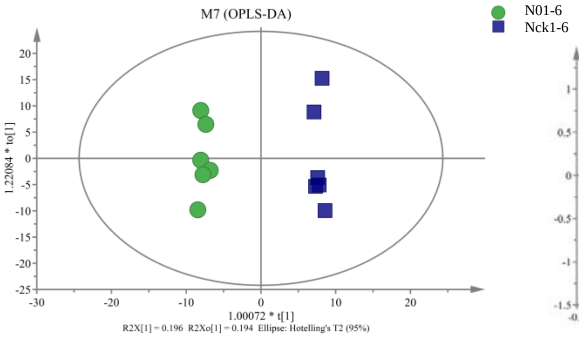

(c)

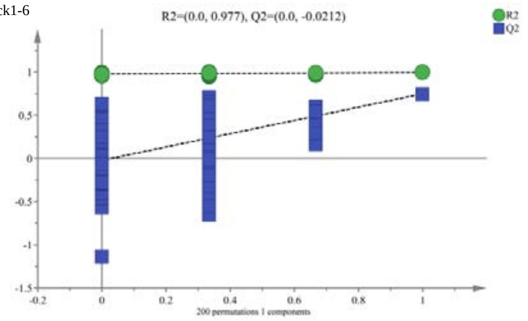

(d)

Supplementary Figure S1 Scores plot of multivariate statistical analysis of accumulated metabolites in N0 and Nck. (a) Principal component analysis (PCA); (b) Partial least squares discriminant analysis (PLS-DA); (c) Orthogonal partial least squares discriminant analysis (OPLS-DA); (d) Response permutation test (RPT) of OPLS-DA model. The X-axis of the PCA score graph represented the first principal component PC1, which was showed with t[1]. The Y-axis represented the second principal component PC2, which was showed with t[2]. N01-6 and Nck1-6 sampling replicates of grains from 6 individual plants. In the score plot, the separation degree of samples from different group can explain the metabolites differences of each group.

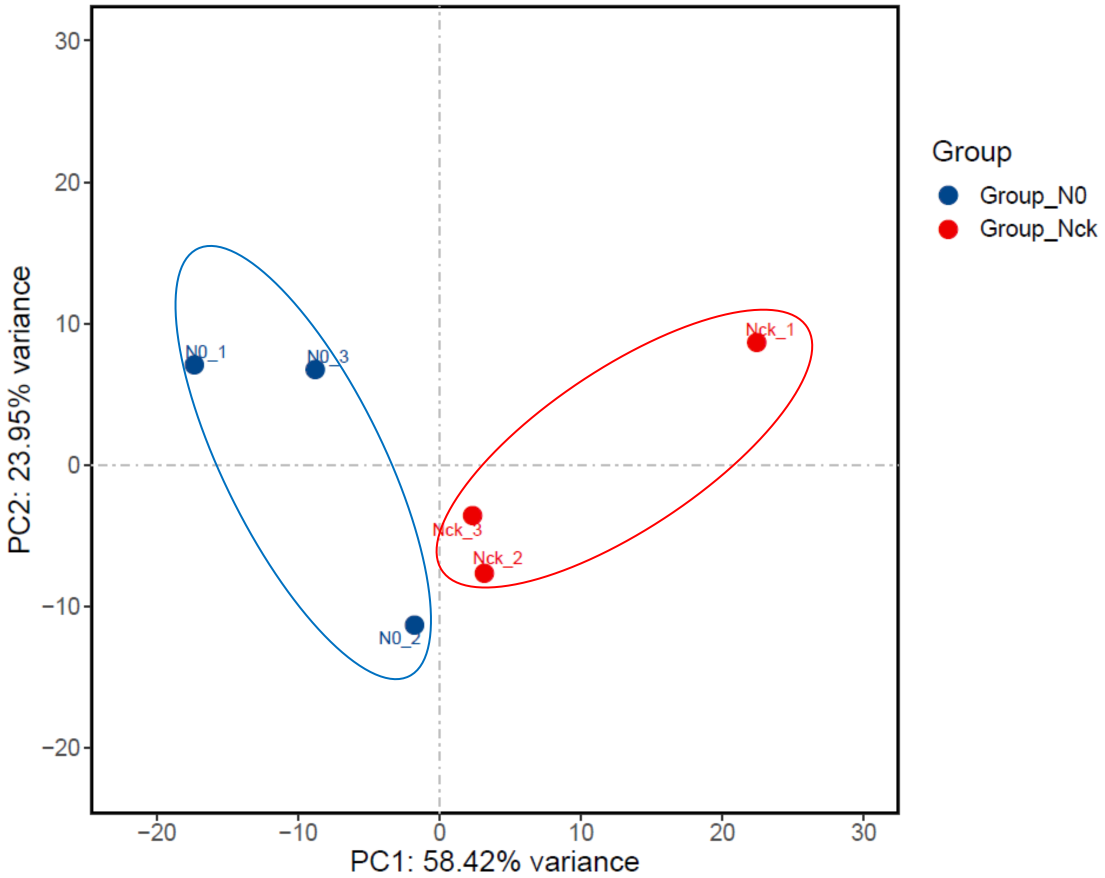

Supplementary Figure S2 Principal component analysis (PCA) of RNA-seq expression in N0 (N-deficient) and Nck (N-sufficient) condition based on FPKM

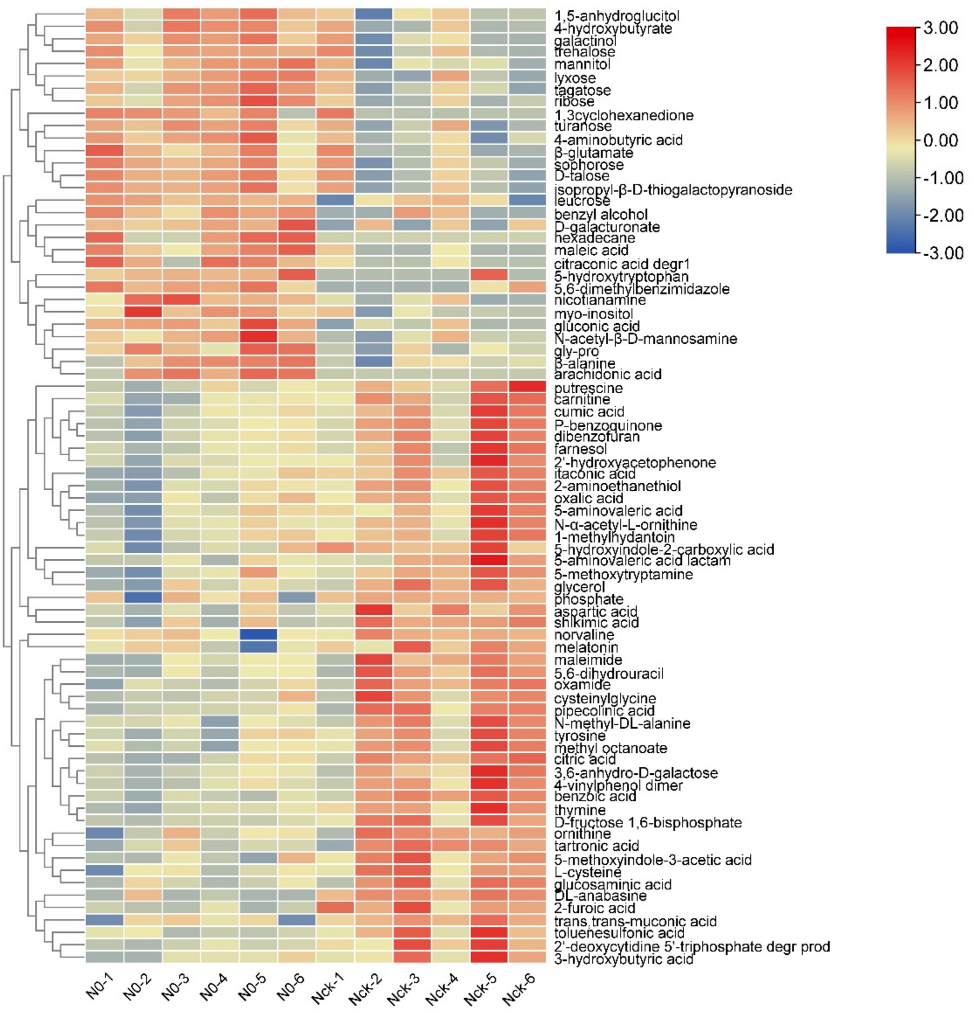

Supplementary Figure S3 The clustering heatmap of content levels for 77 differentially accumulated metabolites (DAMs) under N0 (N-deficient) and Nck (N-sufficient) condition

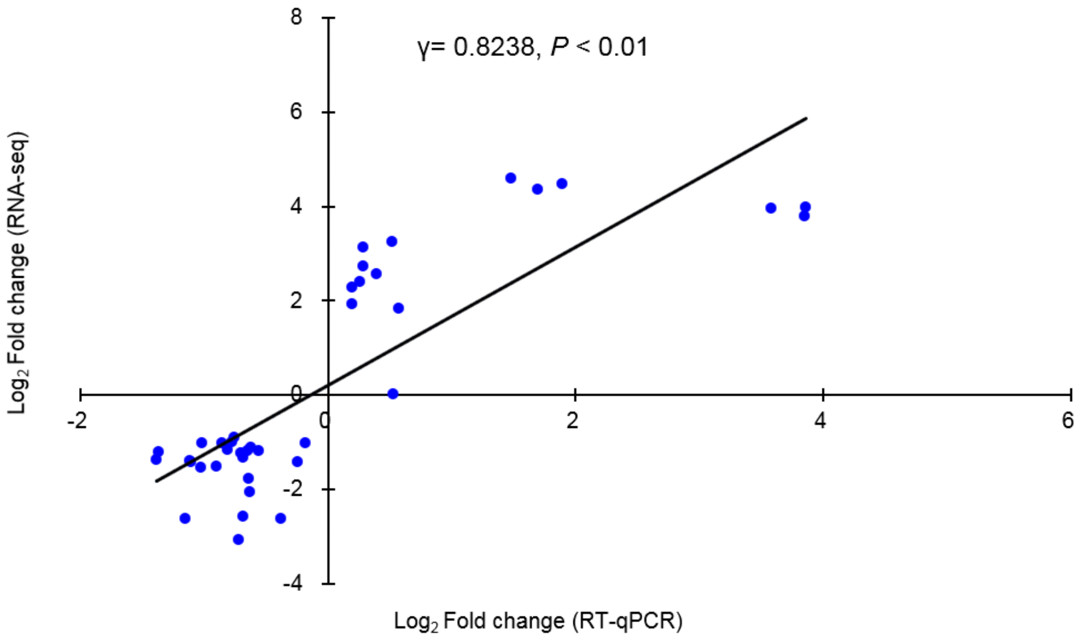

Supplementary Figure S4 Comparison of the $\log_2$ Fold change (Fold change is the value N0/Nck) of 13 selected genes with 3 biological replicates by RNA-seq and RT-qPCR.

Supplementary Table S1. Primers used in qRT-PCR validation

Gene ID	Gene Symbol	Forward primer (5->3)	Reverse primer (5->3)
β-actin	β-actin	TCCAATCTATGAGGGATACACGC	TCTTCATTAGATTATCCGTGAGGTC
TRIAE_CS42_7AS_TGACv1_569206_AA1810290	PETE	AGACCATCACCTTCAAGAAC	TCCTGGGAGATCTTGGAGAC
TRIAE_CS42_2AL_TGACv1_094363_AA0296470	PS I-K	CTCCGGCTCCACAAGATTTA	GGATTTGTGTGCTGTGGATG
TRIAE_CS42_1DL_TGACv1_062185_AA0210150	LHBC	GCCTCGATTACCTTGGAAAC	CTTCAACCAGACCCATGAGA
TRIAE_CS42_2BL_TGACv1_129961_AA0400500	PORA	CACCTTCTCCTCGCTTTAC	CCTTGGTGACGAACTTCTG
TRIAE_CS42_5DL_TGACv1_437406_AA1466150	OsI_012078	GCCCGTAGAGAACCTCTT	TGGACGGACAGATGGCTTA
TRIAE_CS42_U_TGACv1_712376_AA2166870	HSFB2A	TTGACAGGTGGGAGTTCG	TCCCTTCCGCCTCTGTATC
TRIAE_CS42_4DS_TGACv1_362256_AA1178310	CBL3	TTGATAAGACTTTCGAGGAGGC	AGGGATGTCTGAGAACTAGAT
TRIAE_CS42_4BL_TGACv1_321697_AA1064240	HSP70	AGAACGCCCTGGAGAACTA	TCTTCTTGTCGGCCTCTG
TRIAE_CS42_U_TGACv1_640819_AA2075640	PIN9	TCCAAGGACCTCATGAAGC	CCGCCATGTACTCGTAGA
TRIAE_CS42_5BL_TGACv1_404843_AA1312530	mak2	CAGTCAAGCAGCAAGAGC	CCTTGGATGCCATGATCAG
TRIAE_CS42_6AL_TGACv1_471593_AA1511550	WAK5	CAACTGAAGATGAAGGTCGTG	AACTTGAACCCTGCGTAG
TRIAE_CS42_3DL_TGACv1_251332_AA0880190	CDC48C	AGATACTGGAGAGGCTCAC	CCAACGCCTTCAAGTCTG
TRIAE_CS42_6BS_TGACv1_515830_AA1672640	SMC6B	ATCACACTGTGCTGAATGTAT	ATGCAAGAGATTTTCCGACT

Supplementary Table S6. 71 DEGs related to ribosome, DNA and RNA activities, as well as cell cycle under N-deficiency stress

Group	Gene ID	Gene_symbol	log_2FC	Regulation	Description
Sturctural consititute of ribosome	TRIAE_CS42_2AL_TGACv1_093370_AA0278710	RPL4A	2.45	Up	60S ribosomal protein L4-1
	TRIAE_CS42_3DL_TGACv1_250228_AA0864600	RPL18A	1.17	Up	60S ribosomal protein L18-A
	TRIAE_CS42_3DL_TGACv1_252268_AA0888970	RPL20	2.27	Up	50S ribosomal protein L20, chloroplastic
	TRIAE_CS42_7AL_TGACv1_556923_AA1773360	TRIUR3_18443	1.39	Up	hypothetical protein TRIUR3_18443
Ribosome biogenesis in eukaryotes	TRIAE_CS42_1AL_TGACv1_004995_AA0054460	MDN1	1.96	Up	midasin
	TRIAE_CS42_2DL_TGACv1_159051_AA0531450	utp13	1.12	Up	U3 small nucleolar RNA-associated protein 13
	TRIAE_CS42_3AS_TGACv1_212415_AA0700700	rio1	1.07	Up	serine/threonine-protein kinase RIO1
	TRIAE_CS42_3DL_TGACv1_251332_AA0880190	CDC48C	2.72	Up	Cell division control protein 48 homolog C
	TRIAE_CS42_6BL_TGACv1_500458_AA1604970		1.43	Up	uncharacterized protein LOC109783459
	TRIAE_CS42_6DL_TGACv1_529152_AA1717630	naf1	1.67	Up	H/ACA ribonucleoprotein complex non-core subunit NAF1
	TRIAE_CS42_1DL_TGACv1_061207_AA0188840	NLE1	1.35	Up	notchless protein homolog
	TRIAE_CS42_5BS_TGACv1_423949_AA1385270	Os03g0625900	1.12	Up	U3 snoRNP-associated protein-like YAOH
RNA activties	TRIAE_CS42_1BS_TGACv1_049703_AA0160180	NUP107	1.02	Up	nuclear pore complex protein NUP107
	TRIAE_CS42_7BL_TGACv1_577447_AA1875530	NUP50A	1.24	Up	nuclear pore complex protein NUP50A
	TRIAE_CS42_7DL_TGACv1_605020_AA2003890	NRPB8B	1.93	Up	DNA-directed RNA polymerases II, IV and V subunit 8B
	TRIAE_CS42_6DS_TGACv1_543462_AA1740430	RH10	1.70	Up	DEAD-box ATP-dependent RNA helicase 10
	TRIAE_CS42_2DL_TGACv1_159765_AA0542450	MAA3	1.81	Up	Probable helicase MAGATAMA 3
	TRIAE_CS42_7DL_TGACv1_605009_AA2003770	Os06g0526600	1.01	Up	DEAD-box ATP-dependent RNA helicase 31
	TRIAE_CS42_6AL_TGACv1_471618_AA1511850	Os02g0795900	1.00	Up	DEAD-box ATP-dependent RNA helicase 1
	TRIAE_CS42_2AS_TGACv1_114976_AA0370310	Os07g0647900	1.08	Up	DEAD-box ATP-dependent RNA helicase 57
	TRIAE_CS42_7DS_TGACv1_625521_AA2065400	SYD	1.75	Up	Chromatin structure-remodeling complex protein SYD
	TRIAE_CS42_5BL_TGACv1_404284_AA1293840	SUV3	1.07	Up	ATP-dependent RNA helicase SUV3, mitochondrial
	TRIAE_CS42_5DL_TGACv1_433145_AA1403600	U2AF65A	1.07	Up	splicing factor U2af large subunit A-like isoform X1

	TRIAE_CS42_7DS_TGACv1_622146_AA2033840	BRR2A	1.13	Up	DExH-box ATP-dependent RNA helicase DExH12
	TRIAE_CS42_5AL_TGACv1_374601_AA1204150	WTF9	1.01	Up	Protein WHAT'S THIS FACTOR 9, mitochondrial
	TRIAE_CS42_6BS_TGACv1_513512_AA1643950	RBP45	1.11	Up	polyadenylate-binding protein RBP45
	TRIAE_CS42_3AL_TGACv1_195115_AA0644960	mrm1	1.22	Up	Mitochondrial rRNA methyltransferase
	TRIAE_CS42_4AS_TGACv1_308103_AA1025890	At2g16880	1.49	Up	pentatricopeptide repeat-containing protein At2g16880
	TRIAE_CS42_7BL_TGACv1_578267_AA1892140	PCMP-E50	1.36	Up	pentatricopeptide repeat-containing protein At2g37320
	TRIAE_CS42_1DS_TGACv1_081154_AA0258130	VPS60-1	1.71	Up	Vacuolar protein sorting-associated protein 60.1
DNA activities	TRIAE_CS42_2AL_TGACv1_095939_AA0315910	dna2	1.95	Up	DNA replication ATP-dependent helicase/nuclease DNA2
	TRIAE_CS42_4BS_TGACv1_329875_AA1104480		2.77	Up	hypothetical protein TRIUR3_05591
	TRIAE_CS42_6BS_TGACv1_513549_AA1644650	JASON	1.71	Up	protein JASON
	TRIAE_CS42_2AL_TGACv1_095202_AA0308280	LAX2	1.63	Up	Protein LAX PANICLE 2
	TRIAE_CS42_3AL_TGACv1_193661_AA0616880	At3g10140	1.88	Up	DNA repair protein recA homolog 2, mitochondria
	TRIAE_CS42_4AS_TGACv1_306214_AA1004350	RPA1B	1.12	Up	replication protein A 70 kDa DNA-binding subunit B
	TRIAE_CS42_1BL_TGACv1_031515_AA0115400	pfh1	1.31	Up	ATP-dependent DNA helicase pfh1
	TRIAE_CS42_1BS_TGACv1_049674_AA0159400	pfh1	1.35	Up	ATP-dependent DNA helicase pfh1
	TRIAE_CS42_5BL_TGACv1_405283_AA1324030	WDHD1	1.06	Up	WD repeat and HMG-box DNA-binding protein 1
	TRIAE_CS42_6BL_TGACv1_500113_AA1599290	Os02g0805200	1.05	Up	Proliferating cell nuclear antigen
	TRIAE_CS42_3DS_TGACv1_273254_AA0929940	NTO1	1.33	Up	Mst2 complex subunit nto1
	TRIAE_CS42_4AL_TGACv1_289060_AA0964300	DUT	1.87	Up	deoxyuridine 5'-triphosphate nucleotidohydrolase
	TRIAE_CS42_4DS_TGACv1_361176_AA1162740	DUT	1.44	Up	deoxyuridine 6'-triphosphate nucleotidohydrolase
	TRIAE_CS42_6BS_TGACv1_515830_AA1672640	SMC6B	3.91	Up	structural maintenance of chromosomes protein 6B
	TRIAE_CS42_1AL_TGACv1_001426_AA0030330	BRCA1	1.27	Up	protein BREAST CANCER SUSCEPTIBILITY 1 homolog
	TRIAE_CS42_2DS_TGACv1_177481_AA0578260	BARD1	1.32	Up	BRCA1-associated RING domain protein 1
	TRIAE_CS42_7BL_TGACv1_577187_AA1868260	FAS2	1.04	Up	Chromatin assembly factor 1 subunit B
	TRIAE_CS42_5AL_TGACv1_376504_AA1237740	FSM	1.22	Up	Chromatin assembly factor 1 subunit FSM
	TRIAE_CS42_3AL_TGACv1_194491_AA0634010	TH123	1.26	Up	Histone H2B.6

	TRIAE_CS42_U_TGACv1_640700_AA2069590	H2B.2	1.38	Up	histone H2B.2
	TRIAE_CS42_1DL_TGACv1_064141_AA0232850	hh4	1.13	Up	Histamine H4
	TRIAE_CS42_2BL_TGACv1_133205_AA0441830	HMGB6	1.70	Up	high mobility group B protein 6
Cell cycle	TRIAE_CS42_U_TGACv1_640835_AA2076520	STAG1	4.34	Up	Cohesin subunit SA-1
	TRIAE_CS42_3AL_TGACv1_196057_AA0656880	SMC2-2	1.07	Up	structural maintenance of chromosomes 2
	TRIAE_CS42_5BL_TGACv1_408575_AA1363750	SYN3	1.81	Up	sister chromatid cohesion 1 protein 3
	TRIAE_CS42_5DS_TGACv1_456541_AA1473500	CYCA3-2	1.77	Up	cyclin-A3-2
	TRIAE_CS42_7BL_TGACv1_578270_AA1892210	SYP132	1.54	Up	Syntaxin-132
	TRIAE_CS42_3AL_TGACv1_193729_AA0618670	DRP5A	1.31	Up	dynamin-related protein 5A
	TRIAE_CS42_3B_TGACv1_221031_AA0727440	DRP5A	1.51	Up	dynamin-related protein 5A
	TRIAE_CS42_1DL_TGACv1_062802_AA0220170	MAP65-5	1.23	Up	65-kDa microtubule-associated protein 5
	TRIAE_CS42_4AL_TGACv1_289242_AA0967840	TPX2	1.30	Up	Protein TPX2
	TRIAE_CS42_U_TGACv1_642090_AA2111140	KLC3	1.61	Up	kinesin light chain 3
	TRIAE_CS42_5AS_TGACv1_393918_AA1277190	KIN14R	1.08	Up	kinesin-like protein KIN-14R
	TRIAE_CS42_7BL_TGACv1_578441_AA1894940	TUBB1	1.28	Up	Tubulin beta-1 chain
	TRIAE_CS42_7BS_TGACv1_592328_AA1935710	RAC4	1.33	Up	putative small GTP-binding protein
	TRIAE_CS42_1BL_TGACv1_030848_AA0102230	NET2A	2.38	Up	protein NETWORKED 2A
	TRIAE_CS42_3B_TGACv1_222970_AA0774390	XI-K	1.51	Up	Myosin-17
	TRIAE_CS42_6AL_TGACv1_471715_AA1513370	HIP1	1.76	Up	Probable E3 ubiquitin-protein ligase HIP1
Cell proliferation	TRIAE_CS42_5AL_TGACv1_375628_AA1224770	At3g07870	3.21	Up	F-box protein At1g47340 isoform X1
	TRIAE_CS42_2DL_TGACv1_159374_AA0537360	TIF3I1	3.40	Up	Eukaryotic translation initiation factor 3 subunit I
	TRIAE_CS42_5AL_TGACv1_378415_AA1253190	At3g07870	1.43	Up	F-box protein At3g07870

Supplementary Table S7 The DEGs related to ion uptake and transport, disease resistance, and abiotic stress resistance under N-deficiency stress

Group	Gene ID	Gene_symbol	log_2FC	Regulation	Description
Ion uptake and transport	TRIAE_CS42_3DL_TGACv1_249328_AA0845500	NPF1.2	1.21	Up	protein NRT1/ PTR FAMILY 1.2
	TRIAE_CS42_4DL_TGACv1_346114_AA1155110	NPF1.2	1.79	Up	protein NRT1/ PTR FAMILY 1.2
	TRIAE_CS42_4BL_TGACv1_320961_AA1052590	SULTR1;2	1.87	Up	sulfate transporter 1.2
	TRIAE_CS42_4AS_TGACv1_308266_AA1027080	At1g04770	1.01	Up	protein SULFUR DEFICIENCY-INDUCED 2
	TRIAE_CS42_5BS_TGACv1_423795_AA1383420	BOR2	1.18	Up	probable boron transporter 2
	TRIAE_CS42_2AS_TGACv1_113103_AA0351270	CCC2	1.48	Up	cation-chloride cotransporter 2
	TRIAE_CS42_2AL_TGACv1_093880_AA0288690	COPT6	1.63	Up	copper transporter 6
	TRIAE_CS42_3DS_TGACv1_274537_AA0936010	SLAH1	-1.52	Down	S-type anion channel SLAH1
	TRIAE_CS42_7BS_TGACv1_592974_AA1946890	PHO1-3	-2.67	Down	phosphate transporter PHO1-3
	TRIAE_CS42_3B_TGACv1_221653_AA0746670	TPK1	-1.04	Down	two-pore potassium channel 1
Disease resistance	TRIAE_CS42_1DL_TGACv1_061518_AA0197540	RGA2	1.07	Up	disease resistance protein RGA2
	TRIAE_CS42_2AS_TGACv1_115149_AA0371220	RGA3	1.27	Up	putative disease resistance protein RGA3
	TRIAE_CS42_1BL_TGACv1_031662_AA0118230	RGA3	3.15	Up	putative disease resistance protein RGA3
	TRIAE_CS42_6BS_TGACv1_513906_AA1651870	RGA4	Inf	Up	dsease resistance protein RGA4
	TRIAE_CS42_7AL_TGACv1_557829_AA1786770	RGA5	1.04	Up	disease resistance protein RGA5
	TRIAE_CS42_6BS_TGACv1_515091_AA1667200	RPM1	2.87	Up	disease resistance protein RPM1
	TRIAE_CS42_1DS_TGACv1_080256_AA0244390	RPM1	1.31	Up	disease resistance protein RPM1
	TRIAE_CS42_4AL_TGACv1_289054_AA0964150	RPP13	Inf	Up	disease resistance protein RPP13
	TRIAE_CS42_5AL_TGACv1_376140_AA1232660	RPP13	2.77	Up	disease resistance protein RPP13
	TRIAE_CS42_U_TGACv1_640791_AA2074530	MLO13	1.06	Up	MLO-like protein 13
	TRIAE_CS42_3DS_TGACv1_274058_AA0934550	Os05g0277500	Inf	Up	germin-like protein 5-1

	TRIAE_CS42_2DL_TGACv1_157957_AA0503480		1.11	Up	antimicrobial peptides
	TRIAE_CS42_1DS_TGACv1_080301_AA0245590	RPP13	-3.70	Down	disease resistance protein RPP13
	TRIAE_CS42_7DL_TGACv1_604258_AA1995960	RPP13	-1.21	Down	disease resistance protein RPP13
	TRIAE_CS42_7BL_TGACv1_578155_AA1890310	RPM1	-1.21	Down	Disease resistance protein RPM1
	TRIAE_CS42_2DL_TGACv1_161538_AA0559080	DMR6	inf	Down	Protein DOWNY MILDEW RESISTANCE 6
	TRIAE_CS42_5DS_TGACv1_457075_AA1481960	WIR1A	-4.33	Down	protein WIR1A
Abiotic stress resistance	TRIAE_CS42_5DL_TGACv1_433368_AA1411070	RZ1A	1.01	Up	glycine-rich RNA-binding protein RZ1A
	TRIAE_CS42_7BL_TGACv1_578754_AA1899590	SRC2	1.27	Up	protein SRC2 homolog
	TRIAE_CS42_3DL_TGACv1_253971_AA0895810	LOS1	1.86	Up	elongation factor 2
	TRIAE_CS42_2AL_TGACv1_093460_AA0280570	LTL1	1.30	Up	GDSL esterase/lipase LTL1
	TRIAE_CS42_1DL_TGACv1_061644_AA0200730	At3g50940	2.22	Up	AAA-ATPase At3g50940
	TRIAE_CS42_5AL_TGACv1_375039_AA1214550	BPM3	1.63	Up	BTB/POZ and MATH domain-containing protein 3
	TRIAE_CS42_3DL_TGACv1_249544_AA0851310	BPM1	1.66	Up	BTB/POZ and MATH domain-containing protein 1
	TRIAE_CS42_1BL_TGACv1_033050_AA0136450	LSU2	1.57	Up	protein RESPONSE TO LOW SULFUR 2
	TRIAE_CS42_1AL_TGACv1_001888_AA0036300	IRL	1.52	Up	isoflavone reductase homolog IRL
	TRIAE_CS42_4BS_TGACv1_328221_AA1084870	ZIFL1	2.07	Up	protein ZINC INDUCED FACILITATOR-LIKE 1
	TRIAE_CS42_5BL_TGACv1_406491_AA1346180	FTSH5	4.16	Up	ATP-dependent zinc metalloprotease FTSH 5
	TRIAE_CS42_2DS_TGACv1_179008_AA0603420	HIPP27	1.13	Up	heavy metal-associated isoprenylated plant protein 27
	TRIAE_CS42_6BL_TGACv1_502797_AA1626460	HIPP05	2.11	Up	heavy metal-associated isoprenylated plant protein 5
	TRIAE_CS42_2AS_TGACv1_112831_AA0345900	RDUF1	-2.29	Down	E3 ubiquitin-protein ligase RDUF1
	TRIAE_CS42_2BS_TGACv1_148173_AA0491160	RDUF1	-2.20	Down	E3 ubiquitin-protein ligase RDUF1
	TRIAE_CS42_2DS_TGACv1_177260_AA0571040	RDUF1	-2.42	Down	E3 ubiquitin-protein ligase RDUF1
	TRIAE_CS42_6DL_TGACv1_527070_AA1698080	RHA2A	-1.13	Down	E3 ubiquitin-protein ligase RHA2A
	TRIAE_CS42_2BL_TGACv1_129831_AA0397480	BPM1	Inf	Down	BTB/POZ and MATH domain-containing protein 1

Table S9. The DEGs associated with phytohormones, transcription factors, and protein kinases under N-deficiency stress

Group	Gene ID	Gene_symbol	Log_2FC	Regulation	Description
Ethylene (ETH)	TRIAE_CS42_1BL_TGACv1_030266_AA0084590	CRF6	-2.14	Down	ethylene responsive transcription factor CRF6
	TRIAE_CS42_6DL_TGACv1_527023_AA1697360	ERF054	-1.32	Down	ethylene-responsive transcription factor ERF054
	TRIAE_CS42_1AL_TGACv1_000814_AA0019580	ERF061	-1.29	Down	ethylene-responsive transcription factor ERF061
	TRIAE_CS42_5BL_TGACv1_406063_AA1340110	ERF109	-2.52	Down	ethylene-responsive transcription factor ERF109
	TRIAE_CS42_7AS_TGACv1_569159_AA1808800	RAP2-4	-1.09	Down	ethylene-responsive transcription factor RAP2-4
	TRIAE_CS42_7BS_TGACv1_593422_AA1951390	RAP2-4	-1.28	Down	ethylene-responsive transcription factor RAP2-4
	TRIAE_CS42_U_TGACv1_642197_AA2113160	RAP2-12	-2.24	Down	ethylene-responsive transcription factor RAP2-12
Aminocyclopropane-1-carboxylate (ACC) oxidase (ACO)	TRIAE_CS42_3AL_TGACv1_194843_AA0640520	At1g06620	-1.50	Down	1-aminocyclopropane-1-carboxylate oxidase-like protein 1
auxin (IAA)	TRIAE_CS42_2AL_TGACv1_093659_AA0284740	ARF11	1.73	Up	auxin response factor 11
	TRIAE_CS42_U_TGACv1_640819_AA2075640	PIN9	4.51	Up	probable auxin efflux carrier component 9
Cytokinin (CTK)	TRIAE_CS42_1AL_TGACv1_002153_AA0039220	CKX3	1.39	Up	cytokinin dehydrogenase activity
Transcription factors (TFs)	TRIAE_CS42_1BL_TGACv1_031111_AA0108070	pif1	1.52	Up	transcription factor PIF1, bHLH family
	TRIAE_CS42_5BS_TGACv1_423322_AA1374250	FRS5	1.14	Up	protein FAR1-RELATED SEQUENCE 5, FAR family
	TRIAE_CS42_6DS_TGACv1_542484_AA1721940	FRS5	1.13	Up	protein FAR1-RELATED SEQUENCE 5, FAR family

	TRIAE_CS42_5AL_TGACv1_379331_AA1256080	TIFY10C	2.38	Up	protein TIFY 10c, TIFY family
	TRIAE_CS42_5DL_TGACv1_436873_AA1463710	TIFY10C	1.59	Up	protein TIFY 10c, TIFY family
Serine threonine-protein kinase (STK)	TRIAE_CS42_5BL_TGACv1_406098_AA1340730	PBL9	1.47	Up	serine/threonine-protein kinase PBL9 isoform X2
	TRIAE_CS42_U_TGACv1_645180_AA2143400	PIX13	1.75	Up	serine/threonine-protein kinase PIX13
	TRIAE_CS42_5BS_TGACv1_424668_AA1391180	KIPK1	1.35	Up	serine/threonine-protein kinase KIPK
	TRIAE_CS42_3AS_TGACv1_212415_AA0700700	RIO1	1.07	Up	serine/threonine-protein kinase RIO1-like
Wall-associated receptor kinase (WRK)	TRIAE_CS42_6AL_TGACv1_471593_AA1511550	WAK5	1.98	Up	wall-associated receptor kinase 5
	TRIAE_CS42_6DS_TGACv1_543020_AA1734220	WAK5	1.02	Up	wall-associated receptor kinase 5
	TRIAE_CS42_1BS_TGACv1_050512_AA0172930	WAK2	1.15	Up	wall-associated receptor kinase 5
	TRIAE_CS42_5AL_TGACv1_374728_AA1207650	WAK5	1.67	Up	wall-associated receptor kinase 5
Leucine-rich repeat receptor like kinase (LRR)	TRIAE_CS42_2DS_TGACv1_179217_AA0605290	PEPR1	1.19	Up	LRR receptor-like protein kinase PEPR2
	TRIAE_CS42_6BS_TGACv1_514747_AA1663930	At1g35710	1.65	Up	LRR receptor-like protein kinase At1g35710
	TRIAE_CS42_7AS_TGACv1_570917_AA1842360	GSO1	Inf	Up	LRR receptor-like serine/threonine-protein kinase GSO1
Histidine kinase (HK)	TRIAE_CS42_5BL_TGACv1_404843_AA1312530	mak2	3.92	Up	peroxide stress-activated histidine kinase mak2
	TRIAE_CS42_7BL_TGACv1_578745_AA1899500	AHK1	2.00	Up	histidine kinase 1
